# BDC-YOLO: A Novel Architecture Coupling Dynamic Serpentine Convolutions with Bi-Level Routing Attention for Road Defect Detection

**DOI:** 10.3390/s26165301

**Published:** 2026-08-21

**Authors:** Bo Yang, Hongli Sheng, Chen Geng, Chen Chen, Huiqing Lian

**Affiliations:** 1 School of Emergency Technology and Command, University of Emergency Management, Langfang 065201, China; boyang@ncist.edu.cn (B.Y.); wuqibuyouduo@163.com (H.S.); gengchen@ncist.edu.cn (C.G.); 2 Multi-Scene Water Chain Accident Wisdom Emergency Technology Innovation Center of Hebei, Langfang 065201, China; 3 China Academy of Civil Aviation Science and Technology, Beijing 100084, China; 4 School of Urban Security, University of Emergency Management, Langfang 065201, China

**Keywords:** road damage detection, BDC-YOLO, BRA, DySnakeConv, CARAFE, multi-scale feature fusion

## Abstract

Accurate pavement distress identification is essential for infrastructure maintenance. However, prevailing models frequently underperform in complicated environments due to extreme scale variations, atypical defect geometries, and severe background noise. To mitigate these limitations, this study presents BDC-YOLO, an upgraded detection network built upon the YOLOv8 baseline. The proposed architecture structurally incorporates three specialized mechanisms: Bi-level Routing Attention (BRA) to isolate relevant target features from background artifacts; Dynamic Snake Convolution (DySnakeConv) to capture the topological characteristics of elongated and irregularly shaped cracks; and Content-Aware ReAssembly of FEatures (CARAFE) to minimize information degradation during upsampling and refine multi-scale feature fusion. Evaluated on the RDDChina dataset, BDC-YOLO demonstrates superior accuracy over the baseline and comparative state-of-the-art methods. Specifically, the framework yields a mAP_0.5_ of 88.9%, representing an absolute gain of 4.7% against the standard YOLOv8 model while achieving an inference speed of 175.4 FPS.

## 1. Introduction

The accurate localisation of pavement deterioration is of pivotal importance in infrastructure management, as it ensures road safety, optimises traffic flow and minimises maintenance expenditure. Furthermore, the prompt recognition of surface damage, such as cracks and potholes, empowers highway administrations to conduct proactive interventions, effectively averting the deterioration of minor surface irregularities into severe structural breakdowns [1,2]. As the integration of autonomous monitoring frameworks into highway upkeep initiatives has become increasingly prevalent, the use of computer vision to conduct efficient inspections of road damage has become a focal point in the field of infrastructure management [3]. Furthermore, automated systems can effectively reduce the safety risks associated with labor-intensive inspections and minimize the impact on normal traffic during maintenance [4]. Therefore, developing accurate and robust methods for detecting road defects is a key task in the current management of intelligent transportation infrastructure.

Early research on road defect detection primarily relied on traditional image processing techniques. Azarafza et al. [5] explored detection algorithms based on the differences between defective areas and background pixels; Akagic et al. [6] used Otsu thresholding to evaluate image pixels, binarizing them to extract defective areas; Zhao et al. [7] focused on changes in edge intensity within images, using an improved Canny edge detection algorithm to identify road surface edges and cracks; Jiang [8] et al. proposed a rock surface crack detection algorithm combining the Canny operator with Otsu’s adaptive threshold segmentation. Furthermore, a seed-based region-growing strategy was proposed by Zhou et al. [9], which relies on specific expansion criteria to extract the structural topology of fractures. However, these conventional approaches often fail to accommodate the wide variety of pavement anomalies. This limitation primarily stems from the unpredictable nature of actual road environments, including severe lighting fluctuations and substantial background interference. Consequently, modern investigations increasingly prioritise enhancing the algorithmic precision and resilience in such demanding scenarios.

In recent years, significant breakthroughs have been achieved in object recognition and spatial positioning driven by deep learning frameworks [10]. In the field of image classification, deep convolutional neural networks have been widely used to assign class labels to entire images for the purpose of identifying road defects [11]. To achieve finer-grained localization, hybrid deep learning models based on pixel segmentation have been proposed, which divide images into multiple semantic regions [12]. Despite the efficacy of segmentation techniques in delineating fine-grained anomalies within intricate contexts, such models incur substantial computational overhead. Furthermore, the strict necessity for exhaustive pixel-wise annotations restricts their large-scale implementation in real-world industrial scenarios [13]. In contrast, object detection-based methods address both classification and localization tasks and can handle plant diseases of various scales, making them the current mainstream approach. Functionally, researchers group these models into two distinct branches: two-step frameworks spearheaded by R-CNN and its derivatives [14], alongside unified single-step architectures such as YOLO and SSD [15]. Related research has further confirmed the immense potential of the YOLO series in industrial defect detection [16]; its ability to perform localization and classification in a single step aligns perfectly with the requirements of real-time inspection.

Addressing the limitations of standard object detection models in complex road scenes has necessitated various structural enhancements to the baseline YOLO framework. For example, Huang et al. [17] proposed the Weakly Supervised Patch Label Inference Network (WSPLIN), which effectively alleviated the cost burden of pixel-level annotation in road defect detection; Chen et al. [18] integrated DenseNet modules into an object detection network, achieving good real-time detection performance in road defect recognition tasks; Zhang et al. [19] recently proposed the OBC-YOLOv8 model, which optimised the feature extraction process by introducing full-dimensional dynamic convolution (ODConv), achieving a 1.8% improvement in the mAP@0.5 compared to the baseline on the RDD2022-China dataset. Despite recent advancements in road defect detection, existing frameworks frequently encounter performance bottlenecks in complex real-world scenarios due to three primary challenges. First, road cracks typically possess extreme aspect ratios, high curvature, and discontinuous spatial distributions, necessitating specialized topological feature extraction [20]. Standard square-shaped convolutional kernels often incorporate excessive irrelevant background information when processing such slender structures [21]. Consequently, the continuity of the extracted features is compromised, elevating the false negative rate for fine cracks. Second, real-world road environments present severe background interference, including water stains, shadows, and repair marks, which visually resemble genuine defects [22]. Standard attention mechanisms frequently misassign higher weights to these noise artifacts, thereby escalating the false positive rate through background misclassification. Finally, conventional interpolation-based upsampling during multi-scale fusion tends to discard fine-grained high-frequency spatial details [23]. Such information loss disrupts semantic alignment across feature scales, severely diminishing bounding box localization accuracy and the overall Mean Average Precision (mAP) for small-scale defects, such as minute potholes.

To address the aforementioned technical bottlenecks, and building upon the outstanding baseline performance of YOLOv8 [24,25], this paper proposes an enhanced road defect detection framework—BDC-YOLO. This framework integrates three key customised modules: Firstly, we introduce the Bi-Layer Routing Attention mechanism (BRA) [26], which effectively filters out road surface background noise and enhances the feature responses of actual defects, thereby resolving the issue of feature confusion caused by highly similar pixel intensities [27]. Secondly, we incorporate Dynamic Snake Convolution (DySnakeConv) [28] into the feature extraction network; leveraging topological constraints allows for the refined characterization of geometric contours, which enhances the detection accuracy for complex crack patterns characterized by irregular boundaries and elongated structures. Finally, we adopted the Content-Aware ReAssembly of FEatures module (CARAFE) [29], which effectively overcomes the shortcoming of traditional sampling operations leading to the loss of subtle spatial features, thereby maximising the preservation of semantic coherence for minute defects during the multi-scale feature fusion stage. The main objectives and contributions of this paper are as follows:

1. To address limitations in feature discrimination and detection robustness under complex pavement conditions, we propose BDC-YOLO. This framework incorporates BRA, DySnakeConv, and CARAFE to systematically resolve critical bottlenecks, specifically feature ambiguity, morphological variations, and erroneous detection outputs caused by background interference.
2. To improve feature discrimination, the BRA module is implemented to achieve global-scale adaptive attention via dynamic sparse routing. This architecture effectively filters out background noise, such as zebra crossings and staining, while sharpening responses to fine-grained or poorly defined crack/pothole features. Consequently, the model exhibits improved robustness against texture-induced false alarms.
3. The DySnakeConv (Dynamic Snake Convolution) module has been incorporated into the feature extraction network. Addressing the issue that traditional square convolution kernels tend to introduce redundant background information when processing elongated objects, this module is capable of adaptively adjusting its geometric shape along the object’s boundaries, thereby better aligning with the morphological a priori characteristics of road cracks—which are elongated, curved and extend continuously. This mechanism effectively captures the local topological structure of cracks, optimizing the extraction of fine-grained features, which facilitates the precise localization of both irregular mesh-like cracks and faint linear cracks.
4. To optimize the multi-scale integration process within the neck architecture, the Content-Aware ReAssembly of FEatures (CARAFE) operator is employed. Conventional nearest-neighbor interpolation frequently suffers from fine-grained information decay. Conversely, CARAFE circumvents this limitation by deriving dynamic interpolation kernels directly from the underlying feature semantics. This approach expands the observational context during resolution scaling while keeping computational complexity constrained. Consequently, the structural integrity of small-scale anomalies is retained, yielding superior bounding box regression and recall metrics in the final fused representations.
5. Extensive experiments based on a multinational joint public dataset (RDDChina) demonstrated that BDC-YOLO achieves an mAP_0.5_ of 88.9% whilst maintaining real-time inference speeds, significantly outperforming current baseline models and other state-of-the-art methods in terms of overall performance.

The following sections of this document are organised in a systematic manner. Section 2 presents a concise overview of the baseline YOLOv8 framework, in addition to the existing literature concerning the identification of pavement damage. The subsequent Section 3 provides a comprehensive examination of the architectural components and the specific enhancement mechanisms that have been introduced in the proposed BDC-YOLO. Furthermore, Section 4 discusses the extensive empirical validations that have been performed on public datasets, covering both comparative evaluations and ablation studies. Following this, Section 5 assesses the cross-domain robustness of the model via testing on an external benchmark. Ultimately, the core findings and concluding remarks are summarised in Section 6.

## 2. Related Work

### 2.1. Road Damage Detection

Contemporary deep learning frameworks applied to the identification of pavement anomalies are generally categorised into three distinct paradigms: techniques driven by image classification, pixel-level segmentation, and visual object detection. Rather than pinpointing localized damage, classification approaches aim to assign a singular, global category to the entire visual input. As an illustration, the study by Sari et al. [30] benchmarked diverse convolutional neural network (CNN) models for categorising road damages. However, such methods often fail to pinpoint the exact location of defects and are prone to missing subtle cracks in complex backgrounds. Pixel segmentation-based methods (such as FCT-net proposed by Xiong et al. [3]) provide fine-grained, pixel-level localisation by partitioning images into distinct semantic regions. Although these methods are highly effective at delineating the boundaries of irregular cracks, their inherent high computational complexity and significant demand for pixel-level annotated data have, to some extent, limited their large-scale deployment in practical engineering applications.

Despite the robust precision achieved by two-stage detectors like Faster R-CNN [31], their computational latency frequently prohibits real-time deployment in practical engineering scenarios. To overcome this limitation, industrial pavement monitoring systems have heavily adopted single-stage detectors—most notably the YOLO (You Only Look Once) family—because they seamlessly integrate object categorization and spatial localization into a unified, high-speed operation [17]. For example, Roy et al. integrated DenseNet modules into object detection networks to improve the recognition rate of road surface defects [18]. However, most existing YOLO-based models lack a customised network design tailored to the complex features of road defects; they perform poorly when extracting features from highly irregular, elongated cracks [19] and struggle to effectively distinguish actual defects from visually similar background distractions such as water stains and shadows.

### 2.2. YOLOv8 Object Detection

The YOLOv8 framework was officially launched in 2023. It represents a significant upgrade to single-stage object detection algorithms, having achieved notable breakthroughs particularly in modular architecture design. The model overcomes the limitations of single detection tasks by seamlessly integrating diverse functions such as instance segmentation and pose estimation, endowing it with powerful capabilities to tackle complex computer vision challenges. From an architectural perspective, YOLOv8 adheres to the conventional tripartite detection paradigm. The backbone operates as the primary hierarchical extractor, generating multi-level visual representations from the input data. Subsequently, the neck module deploys a refined Path Aggregation Feature Pyramid Network (PAFPN) to execute bidirectional semantic routing, thereby fusing and enriching cross-resolution features. In the terminal stage, the detection head decodes these aggregated representations to formulate the inferential outputs, comprising category labels, spatial localization coordinates, and associated probability metrics. Furthermore, in order to satisfy the diverse hardware deployment requirements, YOLOv8 is configured with five network scales: n, s, m, l, and x. The parameter counts for these variants span from 1.8 M to 68.9 M, thus allowing practitioners to establish an appropriate trade-off between inference speed and detection accuracy.

This equilibrium between inference efficiency and detection accuracy is driven by both flexible network scaling and targeted architectural optimizations within its internal components [32]. In order to optimise gradient flow distribution and augment feature representations while maintaining low computational overhead, the backbone network incorporates the C2f module. Furthermore, YOLOv8 integrates a decoupled head architecture to isolate classification and regression tasks [33], thereby effectively reducing the operational conflict between spatial localisation and semantic recognition. Concurrently, the adoption of an anchor-free detection paradigm facilitates faster model convergence.

Although the YOLO series and other paradigms (e.g., Transformer-based detectors like RT-DETR) have seen newer iterations recently, YOLOv8 is specifically adopted as the baseline framework for this crack detection task due to three intrinsic architectural advantages. First, its anchor-free mechanism eliminates the necessity of manually designing predefined anchor boxes. This is particularly advantageous for pavement cracks, which exhibit extreme, unpredictable aspect ratios and tortuous trajectories that conventional anchor-based models consistently fail to encompass. Second, YOLOv8 employs a decoupled head, strictly isolating the classification and bounding box regression tasks. This separation is crucial for road defects, as it prevents the spatial regression of highly irregular crack boundaries from interfering with the semantic classification of complex, noise-heavy backgrounds. Finally, compared to more recent architectures (e.g., YOLOv10 or YOLOv11) that incorporate increasingly complex perception mechanisms, YOLOv8 avoids the severe risk of overfitting on low-level texture features typical of road surface datasets. By achieving an optimal equilibrium between robust feature representation (facilitated by the C2f module), computational efficiency, and deployment maturity, YOLOv8 provides the most appropriate and stable foundational architecture for subsequent topological adaptations.

However, whilst YOLOv8 performs exceptionally well in general object detection tasks, when applied directly to the specific domain of road defect detection, it inevitably reveals certain limitations. As some researchers have pointed out, the limited receptive fields of traditional convolutional operations make it difficult to adequately model objects with extreme aspect ratios, irregular topological structures, and severe background interference.

## 3. Proposed Method

This research presents BDC-YOLO, an upgraded pavement distress identification model built upon the YOLOv8 architecture. In practical road inspection scenarios, algorithms frequently encounter severe obstacles, including extreme scale fluctuations of targets, the thin and tortuous geometries of cracks, intense background noise, and suboptimal multi-scale feature fusion. To systematically mitigate these limitations, the baseline network is augmented with three specialised components—BRA, DySnakeConv, and CARAFE—which fundamentally strengthen the capacity for feature extraction and multi-scale representation.

**Figure 1:**
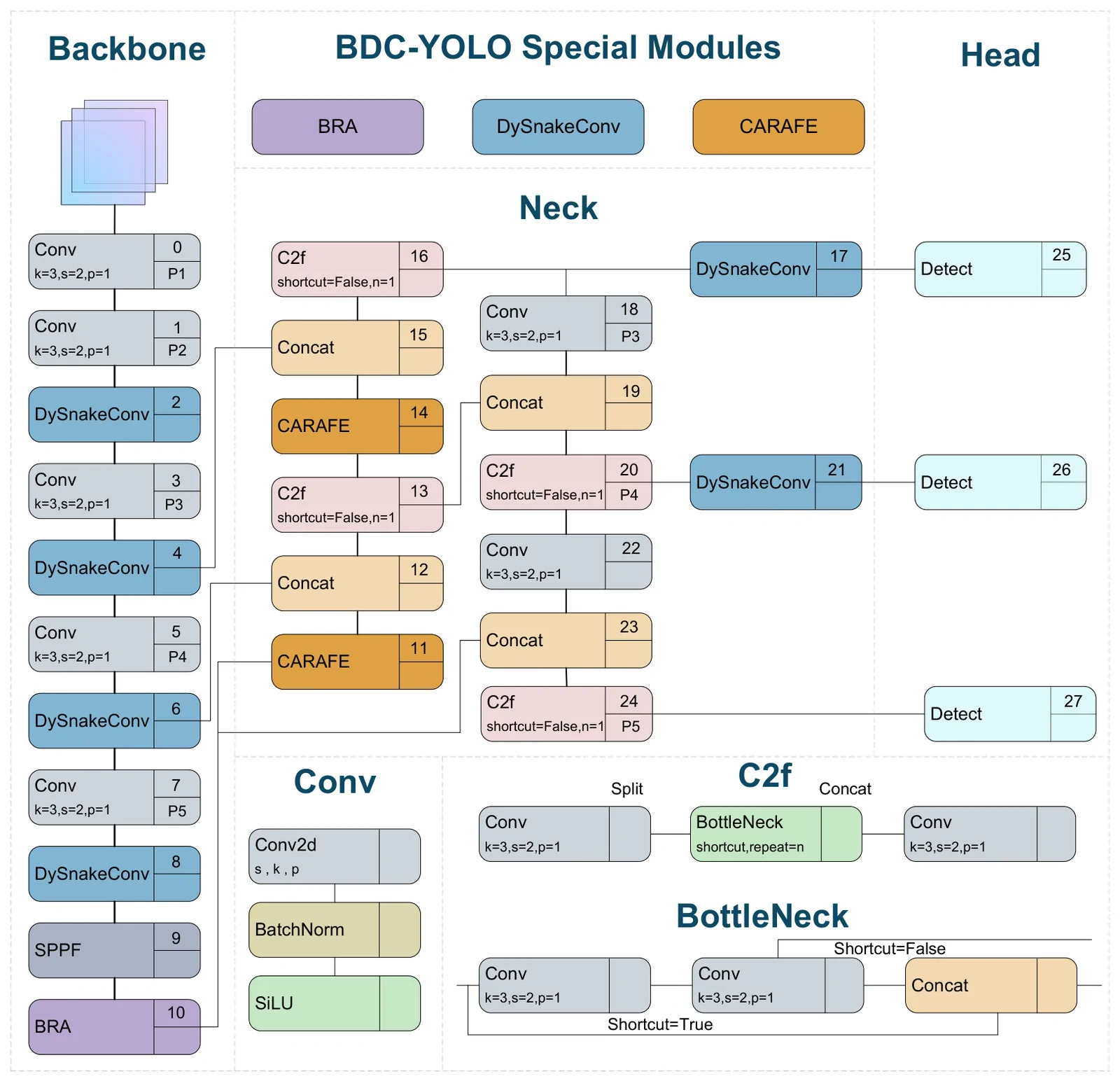
The BDC-YOLO model network architecture.

As demonstrated in Figure 1, the proposed BDC-YOLO is structured upon the foundational Backbone–Neck–Head paradigm of YOLOv8. Within this framework, the Backbone extracts hierarchical visual representations, the Neck aggregates multi-scale features, and the Head computes object classification and localisation outputs. To optimise this baseline, several specific architectural modifications and module selections are implemented.

Initially, a BRA module is integrated into the Backbone. Unlike conventional attention mechanisms (e.g., CBAM or SE) that rely on dense global computations, BRA employs a sparse, region-level routing scheme. This specific selection efficiently isolates critical defect regions while suppressing extensive background noise (e.g., water stains), thereby minimising false-positive rates without imposing excessive computational overhead. Furthermore, DySnakeConv is deployed across the feature extraction and fusion phases. Compared to standard square convolutions or general Deformable Convolutional Networks (DCN) that tend to incorporate irrelevant spatial contexts, DySnakeConv is inherently tailored for tubular structures. This characteristic enables the network to accurately adapt to the slender, tortuous, and irregular morphologies of surface cracks, directly reducing the false negative rate. Finally, the traditional nearest-neighbor upsampling operators within the Neck are replaced by CARAFE. Rather than indiscriminately duplicating pixels, which blurs high-frequency details, CARAFE dynamically generates assembly kernels based on input content. This alternative guarantees superior detail recovery and semantic preservation during multi-scale aggregation, effectively preventing the information degradation of small-scale defects (e.g., minute potholes) and augmenting the overall Mean Average Precision (mAP).

### 3.1. BRA Module

In order to enhance the extraction of features pertaining to critical damage zones within complex road scenes, a BRA module has been integrated into the Backbone. In the context of road defect detection, structural anomalies such as cracks and potholes are characterised by fine textures, blurred boundaries and irregular shapes. Simultaneously, these models demonstrate a high degree of susceptibility to perturbations from complex backgrounds, including shadows, stains, road markings and road surface textures. This interference hinders the network’s capacity to accurately localise authentic defect regions during feature extraction. Moreover, conventional convolutional operations are inherently constrained by their reliance on local receptive fields for feature modelling. This architectural limitation restricts the capture of long-range dependencies, resulting in inadequate activations for defect features and an augmented sensitivity to background noise during the processing of complex backgrounds. Fundamentally, BRA functions as a dynamic sparse attention module driven by a two-layer routing mechanism. Through the synthesis of region-level filtering and fine-grained attention aggregation, this architecture selectively weights target-associated regions while suppressing ambient background noise. This operational optimization subsequently improves both feature representation and overall target detection robustness across complex scenes [26].

The BRA mechanism is fundamentally characterised by its implementation of relevance filtering of input features at a coarse-grained regional level, preceding the execution of fine-grained attention calculations. This preliminary step entails the preservation of exclusively those candidate regions that demonstrate the highest correlation with the active query region. Consequently, this process facilitates token-level attention aggregation within these selected areas. Unlike traditional global self-attention, which establishes dense connections directly between all positions, BRA first constructs semantic relationships between different regions at the regional level and then selects the most relevant regions to participate in subsequent computations via a Top-k strategy. This architecture mitigates the redundant computations typically induced by global attention while sustaining the capacity to capture long-range contextual information. Consequently, computational allocation prioritizes regional information exhibiting high discriminability for object recognition. Applied to road defect detection tasks, this mechanism amplifies feature activations along crack extensions, pothole perimeters, and their adjacent contexts. Concurrently, it attenuates background interference originating from shadows, stains, and road surface textures, systematically bolstering the accuracy and robustness of defect feature extraction.

Formally, given an initial feature map $X\in R^{H\times W\times C}$ (parameterized by spatial height *H*, width *W*, and channel depth *C*), the procedure initiates by partitioning this tensor into distinct non-overlapping regions. Following this spatial division, a linear mapping derives the query, key and value features, formulated as $Q=X^{r}W_{q},\;K=X^{r}W_{k},\;V=X^{r}W_{v}$. Within these transformations, $X^{r}$ corresponds to the regional feature representations post-partitioning, while the components $W^{q}$, $W^{k}$, and $W^{v}$ function as trainable weight matrices. Finally, intra-region aggregation is executed to construct region-level query and key embeddings, facilitating the derivation of the inter-region correlation matrix according to Equation (1):

(1) $$A^{r}=Q^{r}{\left(K^{r}\right)}^{T}$$

Denoting the inter-patch semantic association magnitude as $A_{r}$, the architecture utilizes a Top-k operation to filter and retain the highest-affinity candidate regions per query unit. The outcome of this selective mapping is the instantiation of a routing index matrix, the precise definition of which is detailed in Equation (2):

(2) $$I^{r}=\mathrm{topkIndex}\left(A^{r}\right)$$

Structurally, an individual row within $I_{r}$ encodes the top-*k* region indices that exhibit the strongest correlation with the active query region. Following the derivation of the routing index matrix, representations tied to the identified candidate regions are sampled from both key features and value features. This targeted extraction produces the aggregated key-value features, as mathematically formulated in Equation (3):

(3) $$K^{g}=\mathrm{gather}\left(K,I^{r}\right),\;V^{g}=\mathrm{gather}\left(V,I^{r}\right)$$

Here, $K_{g}$ and $V_{g}$ represent the key features and value features, respectively, aggregated following regional routing. Subsequently, fine-grained attention calculations are performed within the candidate regions, and the output of the BRA is obtained by combining these with a local context enhancement term, as shown in Equation (4):

(4) $$O=\mathrm{Attention}\left(Q,K^{g},V^{g}\right)+\mathrm{LCE}\left(V\right)$$

Here, Attention(·) denotes the attention aggregation operation, whilst LCE(V) denotes the local context enhancement term, which is typically parameterised using deep convolutions to supplement local neighbourhood information [34]. Through the above process, BRA transforms the original globally dense attention modelling into a region-filtered sparse attention computation, enabling the network to capture discriminative features related to disease targets more effectively whilst reducing redundant computations.

**Figure 2:**
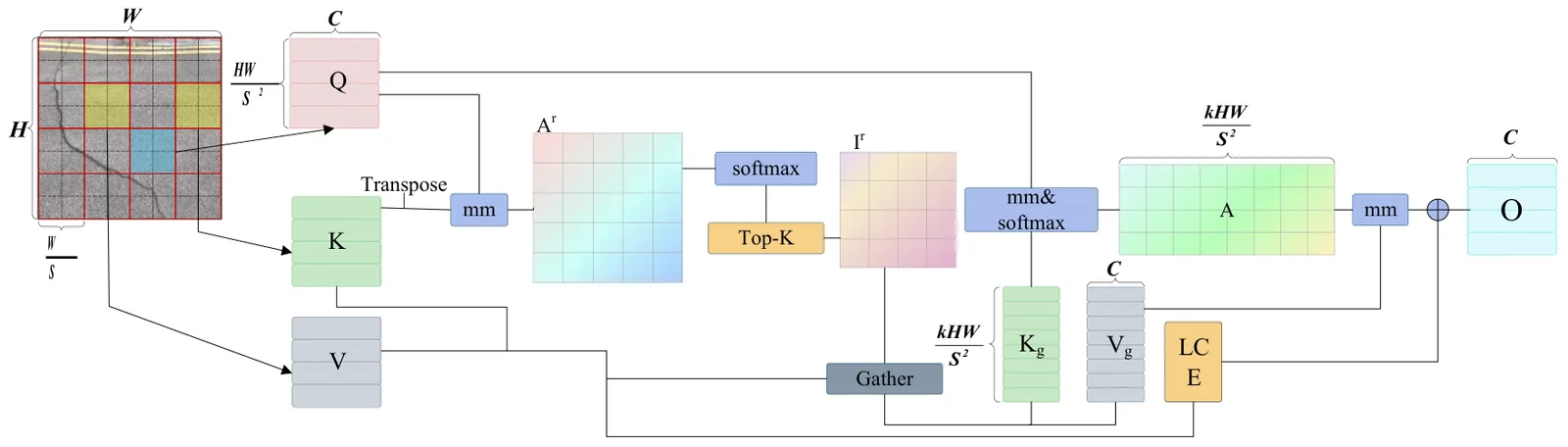
BRA module structure.

The overall structure of the BRA module is shown in Figure 2. The input features are first divided into multiple non-overlapping regions, and three sets of features—query Q, key K and value V—are generated separately; Subsequently, an association matrix $A_{r}$ is computed based on region-level correlations between queries and keys, and a routing index matrix $I_{r}$ is generated via a Top-k operation; building on this, a Gather operation is used to extract information on corresponding candidate regions from the original key-value features, yielding the aggregated $K_{g}$ and $V_{g}$, after which attention calculations and local context enhancement are applied to produce the final features O. This structural diagram intuitively illustrates the BRA module’s processing workflow of ‘region-level filtering—candidate region aggregation—fine-grained attention enhancement’. In order to optimise feature representation, the BRA module is integrated into the deep feature extraction stages of the backbone network. By leveraging the semantic richness inherent in deep feature maps, this implementation facilitates the modelling of long-range dependencies between localized defects and their surrounding context. Such architectural refinement amplifies feature responses corresponding to structural anomalies—such as cracks and potholes—while simultaneously attenuating spurious activations arising from background textures or non-target regions. Consequently, the incorporation of BRA into the backbone network yields high-level semantic features with superior discriminative power, ultimately optimising the accuracy and robustness of road defect detection during the subsequent feature fusion phase.

### 3.2. DySnakeConv Module

For the precise characterisation of slender, curved, and continuously cracked structures, a dynamic snake convolution module is integrated into the feature extraction phase. Evolving from the principles of deformable convolution, the DySnakeConv architecture [28] functions by imposing topological geometric constraints upon the kernel deformation process. This methodology dictates that the receptive field expands adaptively in alignment with the directional trajectory of the target structure, thereby yielding a highly optimized mechanism for feature extraction from slender, continuous targets [35]. For targets such as road cracks, which exhibit distinct directionality, irregular shapes and complex local texture variations, this mechanism helps to suppress feature shifts caused by invalid deformation, preserve richer spatial details, and improve localisation accuracy and feature representation capabilities in complex backgrounds.

**Figure 3:**
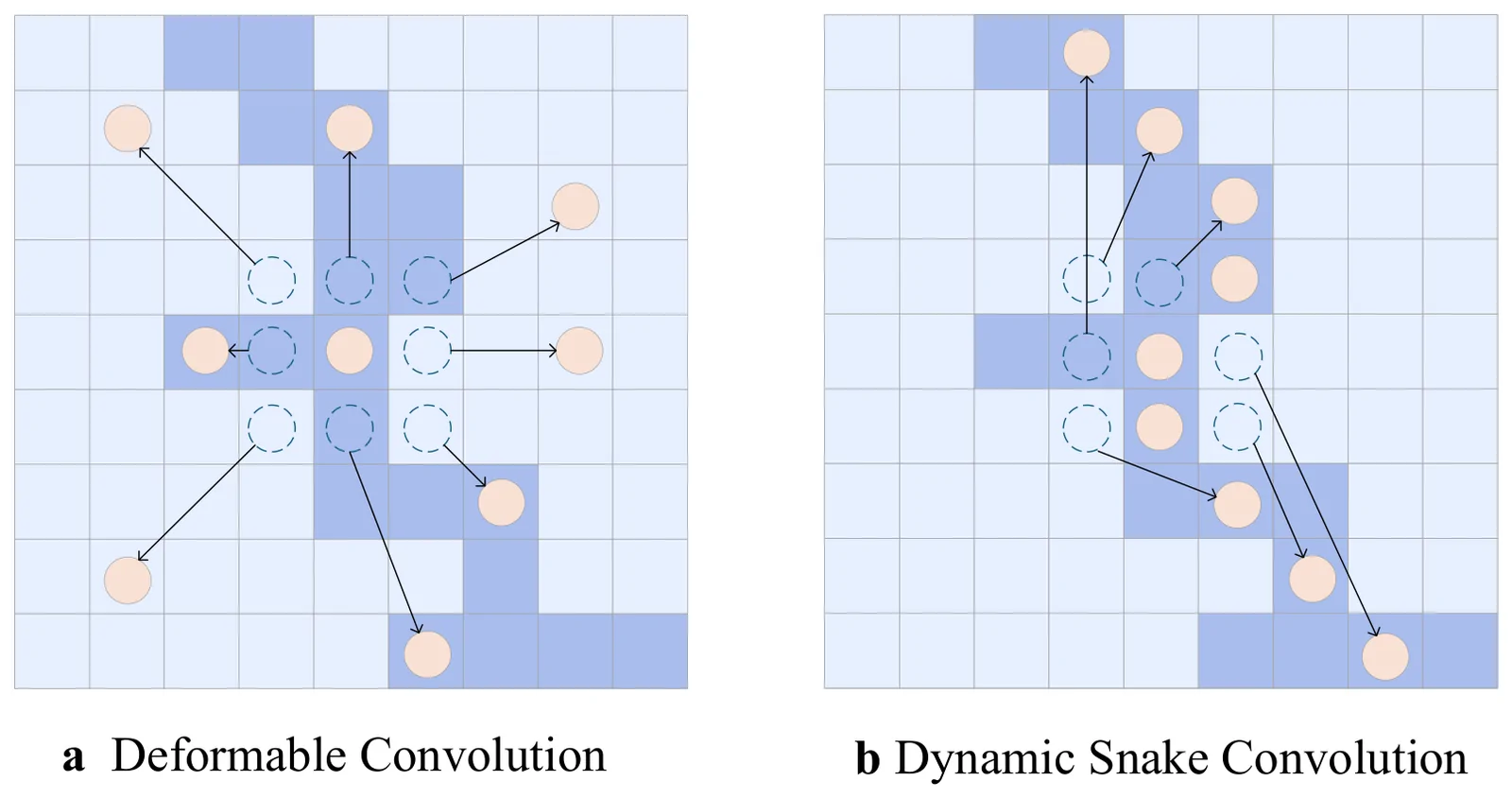
Convolution Kernel Learning Variation Diagram: (**a**) deformable convolution; (**b**) dynamic serpentine convolution.

Figure 3 illustrates the differences between deformable convolution and DySnakeConv during the learning process of the convolution kernel. As shown in Figure 3a, deformable convolution is capable of freely adjusting the sampling positions according to the shape of the object, and is therefore highly adaptable to geometric changes. However, when processing elongated continuous structures such as cracks, some of its sampling points may deviate from the target region or even extend beyond the object’s boundaries, thereby weakening its ability to model the overall morphology of the object. In contrast, as shown in Figure 3b, DySnakeConv incorporates a continuity constraint during the transformation of the convolution kernel, enabling the sampling positions to unfold gradually along the contour of the target. This process enhances the representation of the continuity of elongated structures whilst retaining the convolution kernel’s adaptive deformation capability, thereby addressing the shortcomings of conventional deformable convolution in modelling such targets [28].

The design of the convolutional kernels for DySnakeConv is based on the standard two-dimensional 3 × 3 convolutional structure [36]. Let the centre of the convolutional kernel be at $K_{i}=\left(x_{i},y_{i}\right)$; when the kernel is unfolded along the x-direction, its sampling positions are as shown in Equation (5):

(5) $$K_{i\pm c}=\left\{\begin{array}{c} \left(x_{i+c},y_{i+c}\right)=\left(x_{i}+c,y_{i}+\sum_{i}^{i+c}\Delta y\right),\\ \left(x_{i-c},y_{i-c}\right)=\left(x_{i}-c,y_{i}+\sum_{i-c}^{i}\Delta y\right), \end{array}\right.$$

In this mathematical formulation, $K_{i\pm c}$ dictates the horizontal spatial coordinate of the convolution kernel, while *c* quantifies the discrete stride separating the evaluated location from the kernel’s geometric centre. Furthermore, $K_{i\pm c}$ also characterises the corresponding deformation offset mapped along the y-axis. Extending this identical topological rule to vertical kernel variations yields the positional mapping detailed in Equation (6):

(6) $$K_{j\pm c}=\left\{\begin{array}{c} \left(x_{j+c},y_{j+c}\right)=\left(x_{j}+\sum_{j}^{j+c}\Delta x,y_{j}+c\right),\\ \left(x_{j-c},y_{j-c}\right)=\left(x_{j}+\sum_{j-c}^{j}\Delta x,y_{j}-c\right), \end{array}\right.$$

Here, Δx represents the deformation offset of the convolution kernel in the x-direction. As can be seen from Equations (5) and (6), DySnakeConv does not perform completely free sampling point shifts within a two-dimensional plane; rather, it maintains regular stepping in one direction whilst achieving continuous deformation in the other direction through the gradual accumulation of the offset. Accordingly, the spatial configuration of the convolution kernel achieves rigorous alignment with the topological distribution characteristics inherent to slender structures (e.g., cracks), concurrently preserving its adaptive capability. Given that the spatial coordinates of the convolution kernel frequently shift to non-integer coordinates following dynamic changes, the execution of feature sampling necessitates bilinear interpolation. This continuous mapping procedure is formalized in Equation (7):

(7) $$K=\sum_{K^{\prime }}B\left(K,K^{\prime }\right)\cdot K^{\prime },$$

Here, *K* denotes the decimal coordinate position of the convolution kernel, whilst $K^{\prime }$ denotes the adjacent integer positions surrounding it. The bilinear interpolation kernel function is given by Equation (8):

(8) $$B\left(K,K^{\prime }\right)=b\left(K_{x},K_{x}^{\prime }\right)\cdot b\left(K_{y},K_{y}^{\prime }\right).$$

In this calculation, the operator *B* corresponds to the bilinear interpolation function, with *b* serving as the one-dimensional linear interpolation kernel. Through this interpolation process, DySnakeConv is able to obtain feature responses at non-integer positions based on the continuous deformation of the convolution kernel, thereby providing a more detailed description of the crack boundary and its local geometric variations.

**Figure 4:**
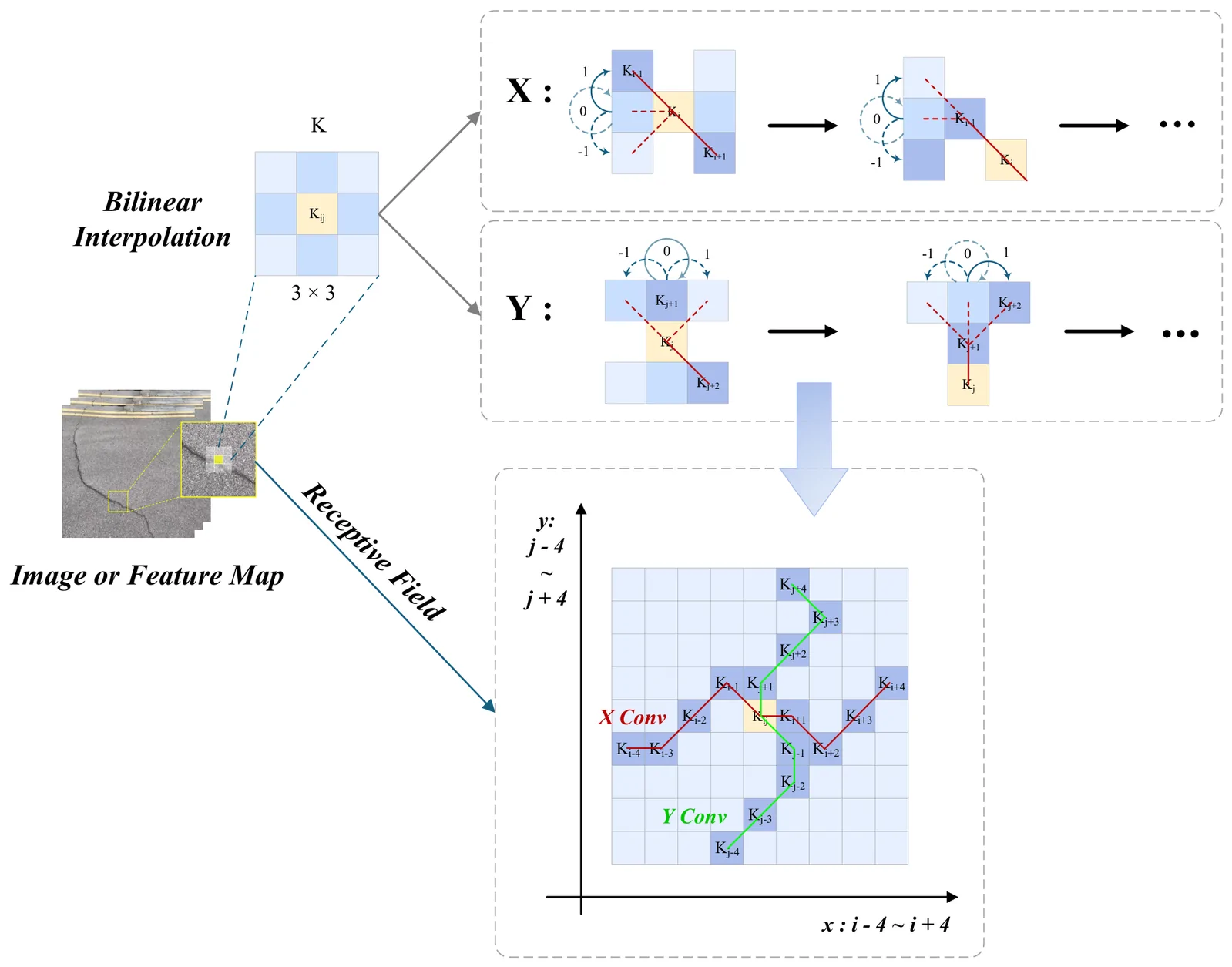
Calculation process of Dynamic Snake Convolution.

Guided by the established principles, the feature extraction stage incorporates DysnakeConv to optimize the geometric modeling of crack targets across diverse orientations and varying morphologies. As delineated in the computational pipeline of Figure 4, the convolution kernel is systematically unfolded along both the x- and y-directions. Fusing these directional expansions with standard convolution operations facilitates a precise extraction of the continuous extension characteristics inherent to crack structures, ultimately yielding an augmented and comprehensive feature representation. For longitudinal, transverse and curved cracks commonly found in road surfaces, this method improves feature extraction performance across the board, thereby providing the subsequent detection head with more accurate information characterising the road defects.

### 3.3. CARAFE Up-Sampling Module

To enhance the ability to recover crack edges, slender structures and local continuity information during the feature fusion stage, this paper introduces the content-aware feature reorganisation operator CARAFE [29] into the upsampling process. Feature upsampling is a critical operation in dense prediction tasks such as object detection and semantic segmentation, and its design directly influences the level of detail preserved when high-level semantic features are restored to high-resolution space. Traditional methods, such as nearest-neighbour interpolation and bilinear interpolation, rely on fixed sampling rules and struggle to achieve adaptive recovery based on local content; whilst deconvolution possesses learnable capabilities, its upsampling kernels are typically shared across different spatial locations, resulting in limited adaptability to changes in local structure [37]. In contrast, CARAFE is capable of dynamically generating position-dependent reconstruction kernels based on the contextual information of the input features and performs content-aware reconstruction of local neighbourhood features; it is therefore better suited to feature recovery for objects such as cracks, which are elongated, have blurred boundaries and exhibit strong continuity [29].

**Figure 5:**
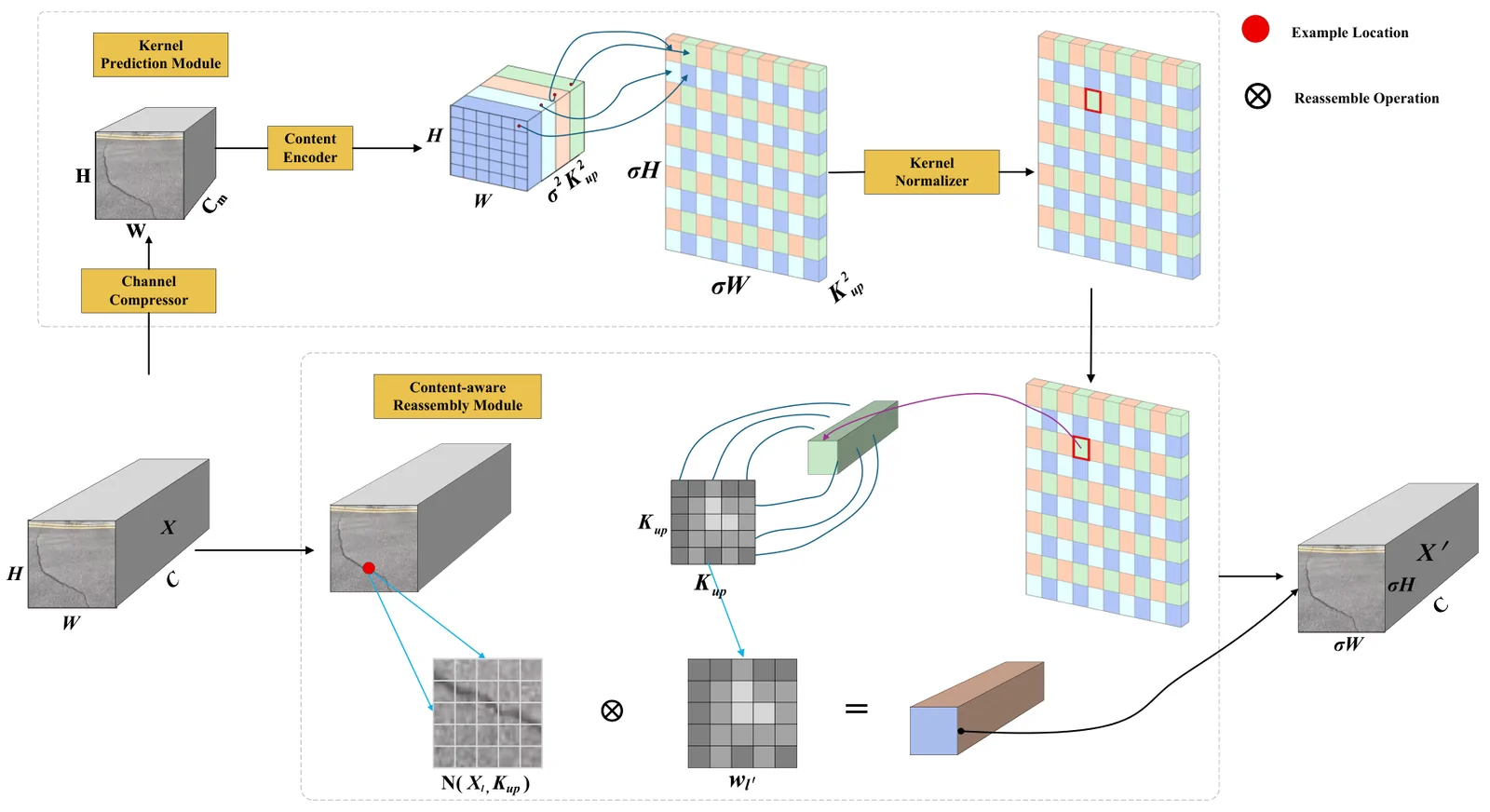
The overall framework of CARAFE.

As depicted in Figure 5, the architectural framework of the CARAFE module is structured around two primary components: the kernel prediction module and the content-aware reorganisation module. The preceding component integrates a channel compressor, a content encoder, and a kernel normaliser to systematically derive a reorganisation kernel tailored to the output spatial coordinate, relying strictly on the local context of the input features. Subsequently, the latter component applies this derived reorganisation kernel to execute a weighted aggregation across the target neighbourhoods within the input feature map, effectively driving the upsampling process.

The upper half of Figure 5 illustrates the process of generating the reconstruction kernel. To control excessive parameters and mitigate computational overhead, the procedure initially subjects the input feature map *X* to channel compression. This mechanism transforms the base channel dimension from *C* to a specified intermediate number of channels, $C_{m}$. Post-compression, the content encoder evaluates the contextual information encompassed by a local receptive field, subsequently deriving the distinct reconstruction kernel parameters designated for each spatial location. Finally, Softmax normalisation is performed via the kernel normaliser to obtain a stable, content-aware reconstruction kernel. The lower half of Figure 5 describes the content-aware reorganisation process: For any position in the output feature map, the corresponding local neighbourhood $N(X_{l},K_{up})$ is first selected from the input feature map; the features within this neighbourhood are then weighted and combined using the reconstruction weight $W_{l^{\prime }}$ corresponding to that position to generate the output response $X_{1^{\prime }}$. As CARAFE employs reconstruction kernels dynamically generated from local content at different spatial positions, it is able to restore spatial resolution whilst more effectively preserving edge, texture and structural continuity information.

The transformation scales an input feature map $X\in R^{C\times H\times W}$ by a specified upsampling ratio *σ*, resulting in the output feature map formulated as $X^{\prime }\in R^{C\times \sigma H\times \sigma W}$. To establish spatial correspondence, each arbitrary target location $l^{\prime }=\left(i^{\prime },j^{\prime }\right)$ within this expanded tensor is mathematically projected back to a source location l=(i,j) on the original grid via the coordinate downscaling $i=\lfloor i^{\prime }/\sigma \rfloor$ and $j=\lfloor j^{\prime }/\sigma \rfloor$. Defining $N(X_{l},K)$ as the *K* × *K* local neighborhood anchored at coordinate *l*, the underlying computational workflow of the CARAFE mechanism proceeds according to Equations (9) and (10):

(9) $$W_{l^{\prime }}=\psi \left(N\left(X_{l},K_{encoder}\right)\right),$$

(10) $$X_{l^{\prime }}^{\prime }=\phi \left(N\left(X_{l},K_{up}\right),W_{l^{\prime }}\right),$$

In this mathematical formulation, ψ(·) acts as the kernel prediction function, mapping the local context of the input features to a distinct reassembly kernel tailored for the target location. The spatial integration is subsequently governed by the content-aware reassembly function ϕ(·), which leverages this predicted reassembly kernel to execute a weighted sum that aggregates the neighboring features. As indicated by Equations (9) and (10), the core idea of CARAFE lies in first predicting a reassembly kernel based on the input content, and subsequently applying this kernel to accomplish local feature reassembly. This establishes a dynamic feature recovery mechanism, which fundamentally distinguishes it from upsampling methods relying on fixed interpolation or shared convolutional kernels.

During the kernel prediction phase, since each spatial location in the input feature map corresponds to $\sigma^{2}$ output locations after upsampling, and each output location requires a reassembly kernel of size $K_{up}\times K_{up}$, the number of output channels for the kernel prediction module is given by Equation (11):

(11) $$C_{up}=\sigma^{2}K_{up}^{2}.$$

To reduce computational complexity, CARAFE first applies a 1 × 1 convolution for channel compression. Subsequently, a content encoder with a convolutional kernel size of $K_{encoder}$ is employed to generate the reassembly kernels, followed by a normalization process. This procedure can be expressed as Equations (12) and (13):

(12) $$\tilde{W}={\mathrm{Conv}}_{K_{encoder}}\left({\mathrm{Conv}}_{1\times 1}\left(X\right)\right),$$

(13) $${\hat{W}}_{l^{\prime }}=\mathrm{Softmax}\left({\tilde{W}}_{l^{\prime }}\right),$$

Here, ${\mathrm{Conv}}_{1\times 1}\left(\cdot \right)$ denotes the channel compression operation, ${\mathrm{Conv}}_{K_{encoder}}\left(\cdot \right)$ denotes the content encoding operation, and ${\hat{W}}_{l^{\prime }}$ represents the normalized content-aware reassembly kernel. Following the Softmax normalization, the sum of the weights within the local kernel at each position equals 1, thereby ensuring numerical stability during the reassembly process. During the content-aware reassembly phase, CARAFE utilizes the normalized reassembly kernel to perform a weighted summation over the corresponding neighborhood in the input feature map, generating the upsampled output response. This expression is formulated as Equation (14):

(14) $$X_{l^{\prime }}^{\prime }=\sum_{n\in N(X_{l},K_{up})}{\hat{W}}_{l^{\prime },n}\cdot X_{n},$$

Here, *n* denotes the sampling location within the input feature map neighborhood $N(X_{l},K_{up})$, ${\hat{W}}_{l^{\prime },n}$ represents the normalized weight assigned to the neighborhood location *n* for the target location $l^{\prime }$, and $X_{n}$ indicates the response value of the input feature at location *n*. As demonstrated by Equation (14), the upsampling process in CARAFE does not rely on fixed interpolation rules. Instead, it achieves feature recovery through local content-driven weighted reassembly. Consequently, it can better preserve information regarding edges, textures, and structural continuity while restoring spatial resolution.

In the domain of road crack detection, crack targets are typically characterised by narrow widths, irregular shapes, low contrast, and weak local texture. Following multiple rounds of downsampling, issues such as blurred edges, loss of detail, and local discontinuities are likely to arise. Incorporating CARAFE into the feature fusion and upsampling stages enables content-aware reorganisation of neighbourhood features within a larger receptive field. This facilitates the retention of high-level semantic information, which in turn enables the restoration of spatial resolution while simultaneously enhancing the network’s ability to characterise crack edges, fine-scale connection regions, and continuous trajectories. Consequently, as a lightweight, dynamic, and content-adaptive upsampling method, CARAFE is better suited for application in the feature restoration process of crack detection networks.

## 4. Experiments

### 4.1. Datasets

Serving as the empirical foundation for this research, the open-source RDD2022 benchmark [34] originates from a global crowdsourced detection challenge. The acquisition of these multi-resolution scenes relied on heterogeneous optical equipment (e.g., smartphones, and high-resolution cameras) deployed via standard vehicles, UAVs, and motorcycles. To ensure comprehensive environmental representation, the multi-angle imagery aggregates records from Asian territories (China, India, Japan), North America (the United States), and European regions (the Czech Republic, Norway). Regarding the labeling schema, the diagnostic targets are formalized under four distinct morphological codes: D00 designates longitudinal cracks; D10 indicates transverse cracks; D20 represents mesh cracks; and D40 is reserved for potholes.

**Figure 6:**
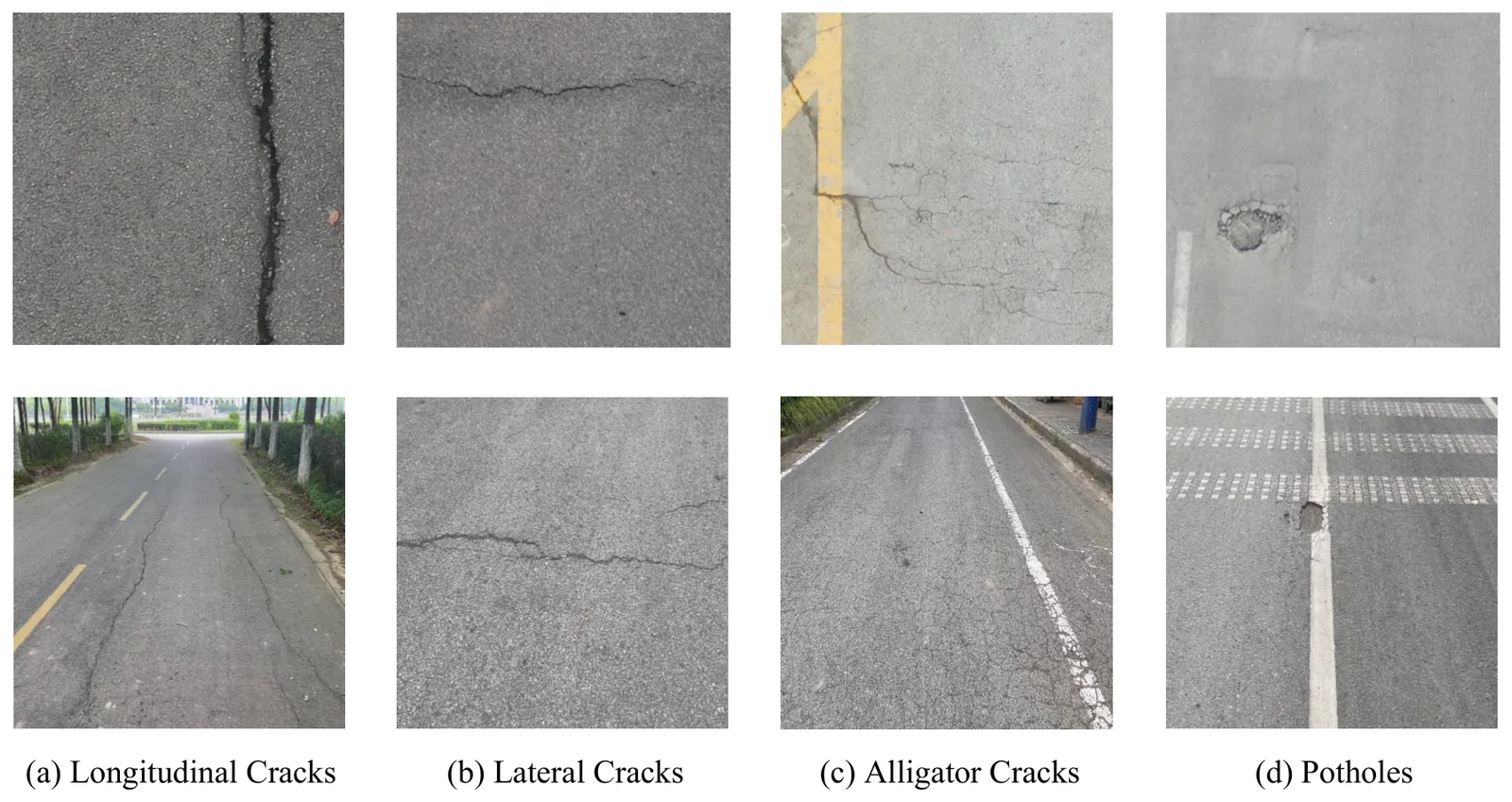
RDDChina: ChinaDrone (**top**) and ChinaMotorBike (**bottom**).

The current research focuses specifically on the Chinese portion of the RDD2022 dataset, encompassing visual data acquired via drones (ChinaDrone) and motorcycle-mounted sensors (ChinaMotorBike), as visualised in Figure 6. Because the source material incorporates distress instances across diverse severity levels, it facilitates highly detailed anomaly detection. During the preprocessing phase, all samples lacking annotations were discarded, and the remaining valid records from both sources were unified into a consolidated dataset designated as RDDChina. Ultimately, this unified corpus was partitioned into training, validation, and testing subsets, allocating 70%, 20%, and 10% of the data, respectively. The exact quantitative breakdown of this configuration is documented in Table 1.

To mitigate the prevalent class imbalance inherent in road defect detection, the Mosaic data augmentation strategy is implemented. During the learning phase, this approach synthesizes a composite frame by stochastically merging four distinct inputs. This specific operation inherently expands the variance in target scales, contextual backgrounds, and spatial positions within an individual batch. Consequently, the occurrence rate of infrequent damage categories is elevated without resorting to the repetitive oversampling of identical samples.

### 4.2. Evaluation Metrics

The primary evaluation criteria for the algorithmic efficacy of the model are precision, recall, and mean average precision (mAP). Higher measurement values indicate greater model accuracy. Precision is defined as the fraction of true positive outcomes out of all positive predictions generated. Conversely, recall is defined as the capability to correctly isolate positive samples from the total volume of existing actual positives. The specific definitions of precision and recall are shown in Equations (15) and (16):

(15) $$\mathrm{Precision}=\frac{\mathrm{TP}}{\mathrm{TP}+\mathrm{FP}}$$

(16) $$\mathrm{Recall}=\frac{\mathrm{TP}}{\mathrm{TP}+\mathrm{FN}}$$

Within these formulations, the quantities of True Positives, False Positives, and False Negatives are parameterized as TP, FP, and FN, in that order. To establish a holistic performance benchmark across multi-class problems inherent to object detection tasks, Mean Average Precision (mAP) is adopted. The exact mathematical derivation for this metric is detailed in Equation (17):

(17) $$\mathrm{mAP}=\frac{1}{C}\sum_{i=1}^{C}{\mathrm{AP}}_{i}$$

Here, *C* represents the total number of categories, and ${\mathrm{AP}}_{i}$ denotes the Average Precision of the *i*-th category.

### 4.3. Experimental Environment and Configuration Parameters

In order to guarantee methodological reproducibility, it is imperative that all training and evaluation phases are executed within a strictly standardised environment. This environment is governed by constant hardware specifications, software dependencies, and hyperparameter configurations. The foundational computational infrastructure comprised a workstation running Ubuntu 20.04, which paired an Intel CPU and 32 GB of RAM with a single NVIDIA RTX 5070 Ti GPU (16 GB VRAM) to manage parallel processing demands. Within the software stack, algorithms were scripted in Python 3.10. Neural network operations were structured through PyTorch 2.9, interfacing directly with CUDA 13 for hardware acceleration. The pipeline was further supported by specific analytical libraries: the Ultralytics module anchored the YOLOv8 architecture, OpenCV executed image pre-processing, Matplotlib generated visual outputs, and Scikit-learn quantified the performance metrics. The specific experimental environment configuration is shown in Table 2.

In order to ensure the reproducibility of experiments and facilitate stable convergence, the global hyperparameters were established through a combination of previous foundational studies, empirical tuning, and validation experiments. Fundamental training mechanisms, including the stochastic gradient descent optimiser and linear warm-up schedules, followed the standard configurations of established previous studies. Furthermore, parameters specific to this detection task were identified via rigorous empirical tuning. The detailed setup involved the network undergoing training for 500 epochs using the optimiser that was previously mentioned. The initial learning rate was set to 0.01 and the batch size was configured to 32, with both values being derived from the validation experiments and the empirical tuning results. In order to mitigate early-stage gradient instability, a linear warm-up strategy was applied during the first 3 epochs. An early stopping mechanism with a patience of 30 epochs was utilised to actively prevent overfitting and determine the optimal training duration. Furthermore, mosaic data augmentation was disabled in the final 10 epochs to refine the model weights on unperturbed data distributions. Additionally, the parameter sensitivity analysis regarding the internal configurations of the proposed modules (such as the routing threshold in the BRA module) is comprehensively detailed in the ablation study (Section 4.5).

### 4.4. Comprehensive Comparison of Different Models

To rigorously evaluate the operational capacity of BDC-YOLO, extensive benchmarks were executed against a diverse selection of state-of-the-art (SOTA) detection algorithms. This baseline pool encompasses the classic two-stage Faster R-CNN, the attention-driven DETR-R50, a customized OBC-YOLOv8 network, and standard mainstream iterations spanning the YOLO series from YOLOv5n up to YOLOv26n. In order to eliminate any potential biases arising from hardware or configuration differences, the entire suite of comparative models was subjected to a standardised retraining protocol. This protocol utilised an NVIDIA RTX 5070 Ti GPU, operating under strictly synchronised hyperparameter constraints.

As synthesized in Table 3, BDC-YOLO consistently outperforms all comparative models. Specifically, it establishes the top performance threshold by securing a Precision of 88.1%, a Recall rate of 82.5%, and a peak mAP_0.5_ score of 88.9%. In object detection tasks, models typically face a strict trade-off between Precision and Recall. However, the quantitative results indicate that our method effectively mitigates this mutual exclusivity, improving the overall detection comprehensiveness while maintaining high localization accuracy.

In comparison with the original baseline YOLOv8n, BDC-YOLO demonstrates a substantial enhancement in overall accuracy (mAP_0.5_) by 4.7 percentage points, accompanied by a notable 6.9 percentage point increase in Recall. Moreover, in contrast to another recent modification, OBC-YOLOv8, our approach exhibits a marked advantage, surpassing it by 9.8 percentage points in Precision and 2.2 percentage points in mAP_0.5_. This substantiates the efficacy of the proposed DySnakeConv and CARAFE modules in extracting and preserving fine-grained topological features of complex road damages.

It is also noteworthy that even when compared to the latest YOLOv26n architecture, BDC-YOLO still maintains a 9.0 percentage point lead in mAP_0.5_ (88.9% vs. 79.9%). This proves that for specific linear and fine-grained targets like pavement cracks, targeted structural innovations are significantly more effective than generalized architectural updates.

Regarding non-YOLO architectures, Faster R-CNN experiences a severe drop in Recall (48.6%), highlighting its inflexibility in capturing small or highly irregular targets. Conversely, DETR-R50 suffers from a drastically collapsed Precision (48.7%), primarily attributed to its fixed object query mechanism without Non-Maximum Suppression (NMS), which inevitably generates an excessive amount of redundant bounding boxes.

In order to ascertain the model’s suitability for practical deployment, it is imperative to consider not only the accuracy of the detection process but also the efficiency of the underlying computational processes. As summarized in Table 3, while models such as YOLOv10n and YOLOv26n leverage streamlined architectures to achieve high throughput exceeding 900 FPS, this gain in speed is accompanied by a decline in detection performance, with mAP_0.5_ values falling below 80%. In contrast, BDC-YOLO integrates specialized modules—DySnakeConv and CARAFE—designed to capture intricate defect topologies. Although this results in a manageable computational cost of 4.09 M parameters and 10.60 GFLOPs, the resulting inference speed of 175.4 FPS remains well above the 30–60 FPS threshold typically required for real-time industrial monitoring. Consequently, this design achieves a favorable balance between detection accuracy and computational overhead, demonstrating that BDC-YOLO is well-suited for real-world pavement inspection.

It is evident that the enhancement of performance exhibited by BDC-YOLO is not solely attributable to empirical results. Rather, this enhancement is predominantly attributed to the structural alignment between the proposed modules and the morphological characteristics of pavement defects.

Conventional detectors utilise rigid square kernels, which invariably encode irrelevant background noise during pavement inspection. In contrast, DySnakeConv employs a deformable, tubular receptive field that dynamically adapts to the slender and tortuous topologies of continuous cracks. This maximises defect-specific feature retention while minimising localized interference, thereby explaining the model’s superiority in distinguishing complex cracks from normal textures.

Furthermore, authentic pavement images often contain confounding factors (e.g., shadows and oil spills) that cause false positives in standard models like YOLOv11 and YOLOv26n. The integration of BRA addresses this vulnerability. Through region-level routing and Top-*k* sparsity constraints, BRA dynamically allocates computational resources to the most salient defect regions. This adaptive filtering significantly strengthens noise robustness, directly contributing to the elevated Precision (reaching 88.1%).

Finally, while competing methods typically utilize nearest-neighbor interpolation that induces spatial degradation, our framework employs CARAFE for multi-scale fusion. By generating dynamic recombination kernels based on input semantics, CARAFE preserves the structural integrity of high-resolution details during upsampling. This content-driven reassembly mitigates information loss, fundamentally driving the improved Recall (reaching 82.5%) for miniature or highly fragmented distresses.

Ultimately, the BDC-YOLO algorithm achieved a new state-of-the-art level of accuracy in the inspection of pavement damage by suppressing background noise and dynamically aligning multi-scale morphological features.

### 4.5. Ablation Experiments

To conduct an in-depth analysis of the effectiveness of each improved component in the BDC-YOLO model and the mechanisms underlying their synergistic effects, this study carried out an exhaustive ablation study on the RDDChina dataset. The experiments covered all possible combinations of the baseline model and the three core modules: (A) BRA, (B) DySnakeConv, and (C) CARAFE. Given that real-world road defect detection tasks require a strict balance between false positives and false negatives, we used mAP_0.5_ as the primary metric for evaluating the model’s overall performance. Additionally, precision, recall, the number of parameters and computational cost (GFLOPs) are reported to facilitate a comprehensive assessment. The detailed quantitative evaluation results are shown in Table 4.

1. **Analysis of the validity of a single module:** The baseline model, YOLOv8n, achieves an mAP_0.5_ of 84.2%. Upon individually incorporating Module A (BRA), B (DySnakeConv), and C (CARAFE), the core metric mAP_0.5_ increases independently to 87.0%, 86.4%, and 85.4%, respectively, demonstrating the individual effectiveness of each module in feature processing. In terms of the underlying mechanisms reflected by the auxiliary metrics, BRA yields the most significant improvement in mAP_0.5_ (an increase of 2.8%). This is primarily attributed to the fact that the bi-level routing attention successfully establishes a global receptive field, effectively filtering out high-frequency noise such as tree shadows, which substantially enhances the Recall by 6.0%. Conversely, DySnakeConv achieves the highest Precision (85.8%) among the single-module configurations. This superiority is credited to its tubular receptive field, which adaptively conforms to the topological trajectory of irregular cracks, thereby avoiding the inclusion of irrelevant background pixels typically introduced by conventional square convolutional kernels.
2. **Synergies arising from the combination of the two modules:** An analysis of the pairwise combination experimental groups (+A + B, +A + C, and +B + C) reveals that their core metric, mAP_0.5_, consistently surpasses that of the individual modules. This demonstrates the absence of feature-dimensional conflicts among the proposed improvement strategies. Notably, the +A + B combination achieves an outstanding mAP_0.5_ of 88.3%. This significant gain in comprehensive performance originates from the strong synergistic effect during the cascaded feature processing. When confronting extremely difficult samples, such as high-contrast road markings or complex geometric textures, BRA acts as a preliminary “semantic filter,” preemptively suppressing the response activations of irrelevant edges. Subsequently, DySnakeConv focuses on extracting genuine crack contours from the refined feature maps. This “purify first, focus later” mechanism successfully breaks the mutually exclusive bottleneck between Precision and Recall, ultimately driving a substantial surge in mAP_0.5_.
3. **Overall performance of the three-module integrated system:** Building upon the dual-module foundation, the complete BDC-YOLO model (+A + B + C), constructed by introducing the CARAFE module, achieves a global maximum mAP_0.5_ of 88.9% (simultaneously attaining a Precision of 88.1% and a Recall of 82.5%). In deep neural networks, although deep features possess rich global semantics, they are highly susceptible to the loss of spatial details during conventional nearest-neighbor upsampling. The CARAFE module completely breaks through the information transmission bottleneck within the Feature Pyramid Network (FPN), achieving seamless alignment between shallow high-resolution details and deep semantics via local content-aware processing. This architectural design effectively compensates for the feature loss of multi-scale targets, such as alligator cracks and minute potholes, and further eliminates background false positives, thereby propelling the comprehensive detection capability (mAP_0.5_) to its peak.
4. **The trade-off between computational cost and accuracy:** Furthermore, we report the parameter count and computational complexity (GFLOPs) in the ablation study to quantify the computational cost and efficiency of each variant. As presented in Table 4, although the incorporation of these enhancement modules results in an increase in the parameter count (from 3.01 M to 4.09 M) and GFLOPs (from 8.1 to 10.6), this additional overhead is deemed acceptable given the significant accuracy gain (a 4.7% improvement in mAP_0.5_). As demonstrated by the recently developed FPS metric in Table 4, the comprehensive BDC-YOLO model continues to exhibit an exceptionally elevated inference speed of 175.4 FPS on an RTX 5070 Ti GPU. This outcome validates the efficacy of the proposed architectural enhancements in enhancing detection accuracy without compromising the real-time operational capacity essential for automated pavement inspection. Specifically, inheriting the one-stage architecture of the YOLO series, BDC-YOLO completes the entire detection process in a single forward pass without requiring complex region proposal networks. Furthermore, because its inference speed significantly exceeds the standard real-time processing threshold (typically 30 FPS), the model is highly feasible for direct deployment on resource-constrained, vehicle-mounted edge sensors, enabling continuous and real-time pavement scanning as the vehicle moves.

Beyond the module-level combinations discussed above, we further investigated the impact of different architectural configurations and internal design choices. Specifically, we conducted targeted ablation studies focusing on the integration strategy of the DySnakeConv module and the parameter sensitivity of the BRA module, as detailed in Table 5.

First, regarding architectural configurations, we explored the optimal insertion position of the DySnakeConv module. Crucially, to avoid sub-optimal isolated designs, this architectural search was conducted in conjunction with the BRA module to ensure holistic co-optimization. We evaluated two distinct integration strategies: deploying DySnakeConv solely in the detection head (Head only) and applying it across both the backbone and the head (Backbone + Head, our finalized architecture). As shown in Table 5, the Head-only strategy achieved an mAP_0.5_ of 86.6%. However, deploying it across both the backbone and the head achieved a superior mAP_0.5_ of 88.3%. This demonstrates that applying DySnakeConv in both the feature extraction and multi-scale fusion stages maintains topological continuity and maximizes its synergistic interaction with the BRA module.

Second, regarding additional design choices, we evaluated the internal routing parameters of the BRA module. We compared the default parameter setting [8, 7] (representing region partition and Top-*k* routing threshold, respectively) against our optimized setting [8, 4]. While the default setting [8, 7] captures broader contextual features, it incurs heavier computational overhead and introduces background noise. In contrast, the [8, 4] configuration enforces a stricter sparsity constraint and aligns with GPU memory access patterns, achieving a higher mAP_0.5_ and improved inference efficiency.

Data outlined within Table 6 and Table 7 substantiate the parameter improvements yielded by the BDC-YOLO approach regarding the overall dataset as well as specific road defect categories. On a macro level, an expansion of 4.7 percentage points is achieved in mAP_0.5_, while precision (P) and recall (R) scale upward by 3.6 and 6.9 percentage points, respectively.

An analysis of the mAP_0.5_ metric across individual defect categories reveals consistent quantitative enhancements for all classifications. Specifically, the “pothole” category demonstrates the maximum gain, with an increase of 10.6 percentage points. This phenomenon is largely attributable to the highly discernible morphological patterns and clear regional boundaries characteristic of potholes, which simplify the feature extraction process.

Additionally, the “alligator crack” and “other corruption” classes witness significant improvements of 4.8 and 3.7 percentage points, respectively. The substantial gain in alligator cracks perfectly demonstrates that their complex mesh-like topological structures are more effectively captured by the dynamic receptive fields of the proposed DySnakeConv module.

It is worth emphasizing that even for the highly challenging “longitudinal crack” and “transverse crack” classes, which are easily disturbed by road joints, the model’s mAP_0.5_ still steadily increases by 2.5 and 2.1 percentage points, respectively.

To summarise, the findings of the present study demonstrate that the architectural modifications integrated into the BDC-YOLO framework facilitate the reliable identification of diverse road damages amidst complex pavement scenarios. Consequently, the proposed model yields quantifiable improvements in both detection accuracy and overall performance.

### 4.6. Comparison of Alternative Modules

To rigorously validate the superiority and necessity of the specific architectural choices in BDC-YOLO, we conducted a comprehensive comparative analysis against representative alternative mechanisms that serve similar functional purposes. The evaluation incorporates precision (P), recall (R), and mAP_0.5_ to provide a multi-dimensional assessment. The comprehensive results are detailed in Table 8.

Regarding the attention mechanism, we compared the proposed BRA with mainstream modules including CBAM, MPCA, and CPCA. While CPCA achieved a commendable mAP_0.5_ of 85.5%, BRA (87.0%) significantly outperformed all alternatives, proving its superior efficacy in establishing global receptive fields and filtering road background noise.

For spatial feature extraction, the proposed DySnakeConv (86.4%) was evaluated against the EIEM module and the efficient Ghost convolution. The results demonstrate that DySnakeConv possesses an inherent advantage in modeling slender, tortuous crack topologies compared to standard or lightweight convolutions.

Finally, in terms of multi-scale fusion, the CARAFE module (85.4%) effectively mitigated information degradation during upsampling, exceeding the performance of alternative structures such as AFPN, CAFM, and BiFPN. These extensive comparative results conclusively justify the selection of the BRA, DySnakeConv, and CARAFE combination as the optimal framework for pavement defect detection.

### 4.7. Detection Visualization and Feature Heatmaps

**Figure 7:**
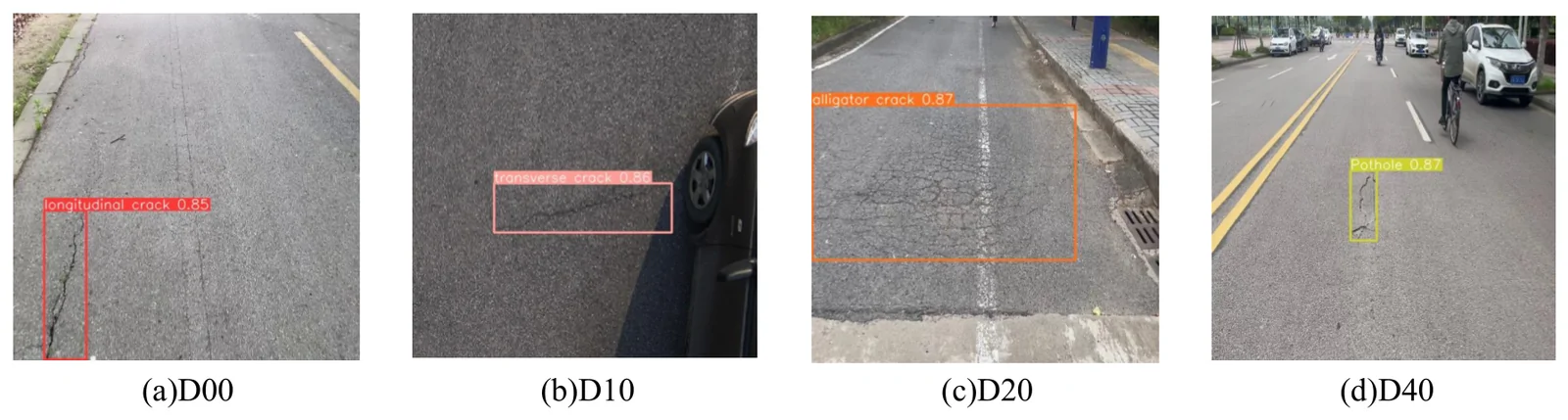
Representative detection examples of road damage across distinct categories.

To facilitate qualitative evaluation, inference was conducted on representative testing instances utilizing both the foundational baseline and the BDC-YOLO architecture. The proposed network’s specific diagnostic outputs are mapped in Figure 7. These visual panels systematically capture the prescribed dataset taxonomies in order: longitudinal cracks (D00); followed by transverse cracks (D10); alligator cracks (D20); and finally, potholes (D40). Based on the observation of these prediction samples across different categories, we can summarize the model’s detection characteristics when processing differentiated damage morphologies.

Regarding regional damages such as D20 alligator cracks and D40 potholes, because they occupy a relatively large pixel area on the road surface and exhibit distinct texture features, the model can effectively extract the feature distributions of these salient damages and accurately localize their physical positions.

As for D00 longitudinal cracks and D10 transverse cracks, these damages visually present fine and elongated morphological characteristics. Due to their finer topological structures and susceptibility to road background interference, conventional models frequently encounter localization difficulties when processing such targets. However, as illustrated in Figure 7a,b, the proposed model demonstrates robust detection capabilities when confronting such linear cracks. Benefiting from the dynamic receptive field mechanism provided by the DySnakeConv module, the model can adaptively adjust the shape of the convolutional kernel according to the geometric trajectory of the cracks. Consequently, it accurately captures the edge features of linear damages amidst complex background noise, effectively mitigating the risk of environmental backgrounds obscuring fine-grained features.

**Figure 8:**
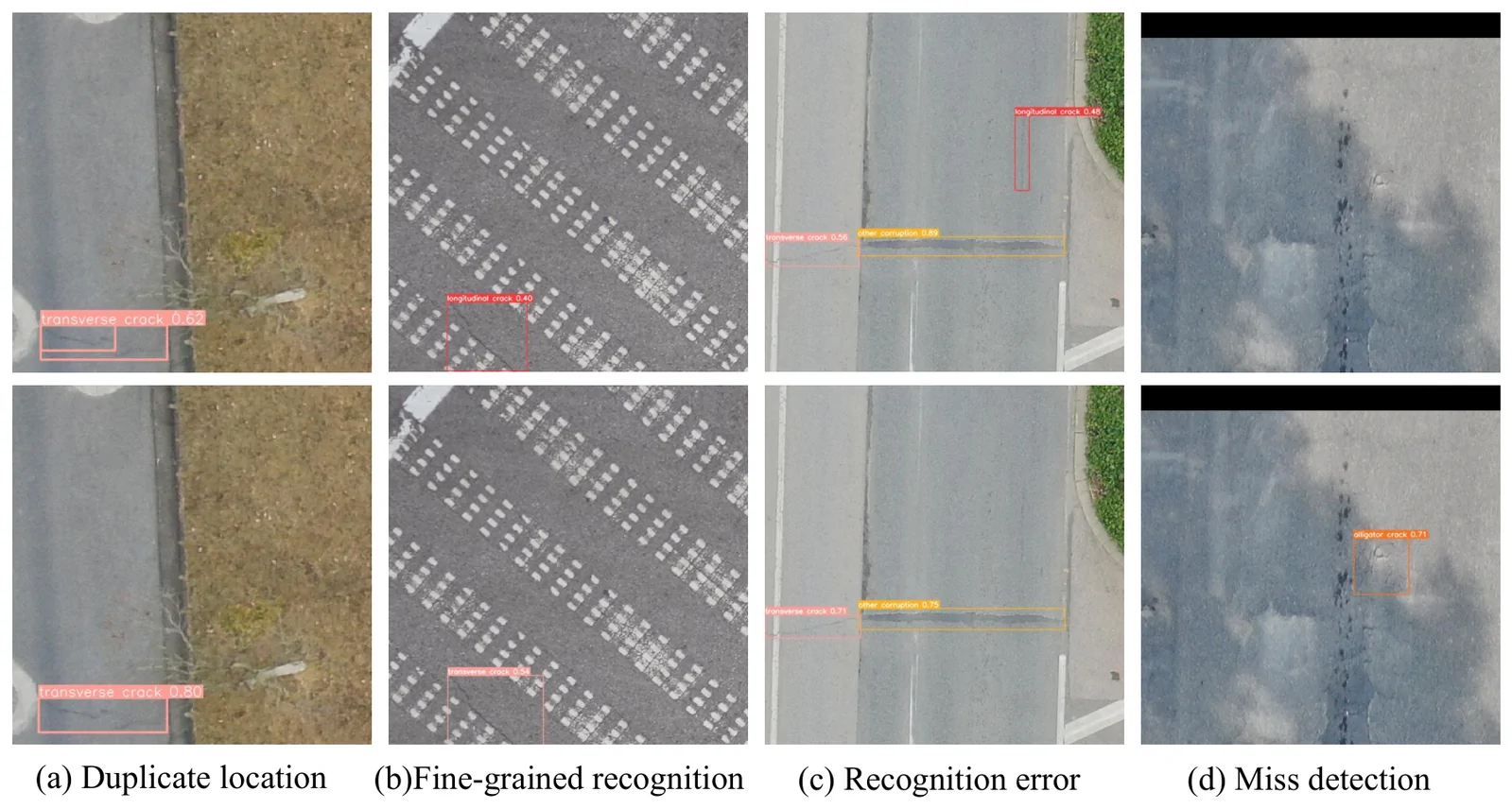
Detection outputs comparison: YOLOv8 (**top**) vs. BDC-YOLO (**bottom**).

As illustrated in Figure 8, a contrast is drawn between the inference outputs of the baseline YOLOv8n (upper panels) and the proposed BDC-YOLO framework (lower panels). The figure provides a comprehensive overview of the generated bounding boxes, defect classifications, and corresponding confidence scores. The customized BDC-YOLO architecture has been shown to yield measurable enhancements in several key operational areas. These include the elimination of redundant localization predictions, the precise encoding of fine-grained local textures, the mitigation of background-induced false positives, and the successful retrieval of previously undetected targets.

Specifically, as illustrated in Figure 8a, in the transverse crack region, the original model generates multiple repetitive and nested bounding boxes. In contrast, the improved model accurately localizes the target and outputs a unique predicted bounding box, effectively eliminating redundant detections.

Figure 8b demonstrates the models’ fine-grained recognition capability in scenarios with severe background interference (e.g., dense road markings). The original baseline model fails to distinguish the fine-grained topological features of the crack from the high-frequency background noise, misclassifying the horizontal crack as a longitudinal one (with a low confidence of 0.40). In contrast, BDC-YOLO effectively penetrates the background interference through its enhanced fine-grained feature perception. It accurately captures the horizontal trajectory of the target, successfully correcting the classification to a transverse crack and boosting the confidence to 0.54, thereby preventing a potential missed detection.

As demonstrated in Figure 8c, a false positive prediction occurs in the baseline output. The original model incorrectly processes the background region on the right side, which comprises a normal road seam and curb edge, as foreground, subsequently classifying it as a longitudinal crack. The upgraded framework eliminates this misclassification by means of the embedded attention mechanism, which suppresses irrelevant high-frequency interference, thereby restricting the network from categorising background elements as road damage, effectively filtering out background outliers without the need for external post-processing refinement.

Figure 8d illustrates the breakthrough of the improved model in recovering missed targets. In the low-contrast vehicle shadow area, the original model fails to detect the hidden crack target. Conversely, the enhanced BDC-YOLO model overcomes the illumination interference and accurately predicts the damage overlooked by the original model.

In summary, the qualitative comparisons of the bounding box outputs confirm that BDC-YOLO effectively resolves typical detection bottlenecks, including redundant localization, fine-grained misclassification, false positives, and missed detections. While these results validate the model’s superiority at the final prediction level, exploring the internal feature representations that drive these accurate decisions is equally essential.

**Figure 9:**
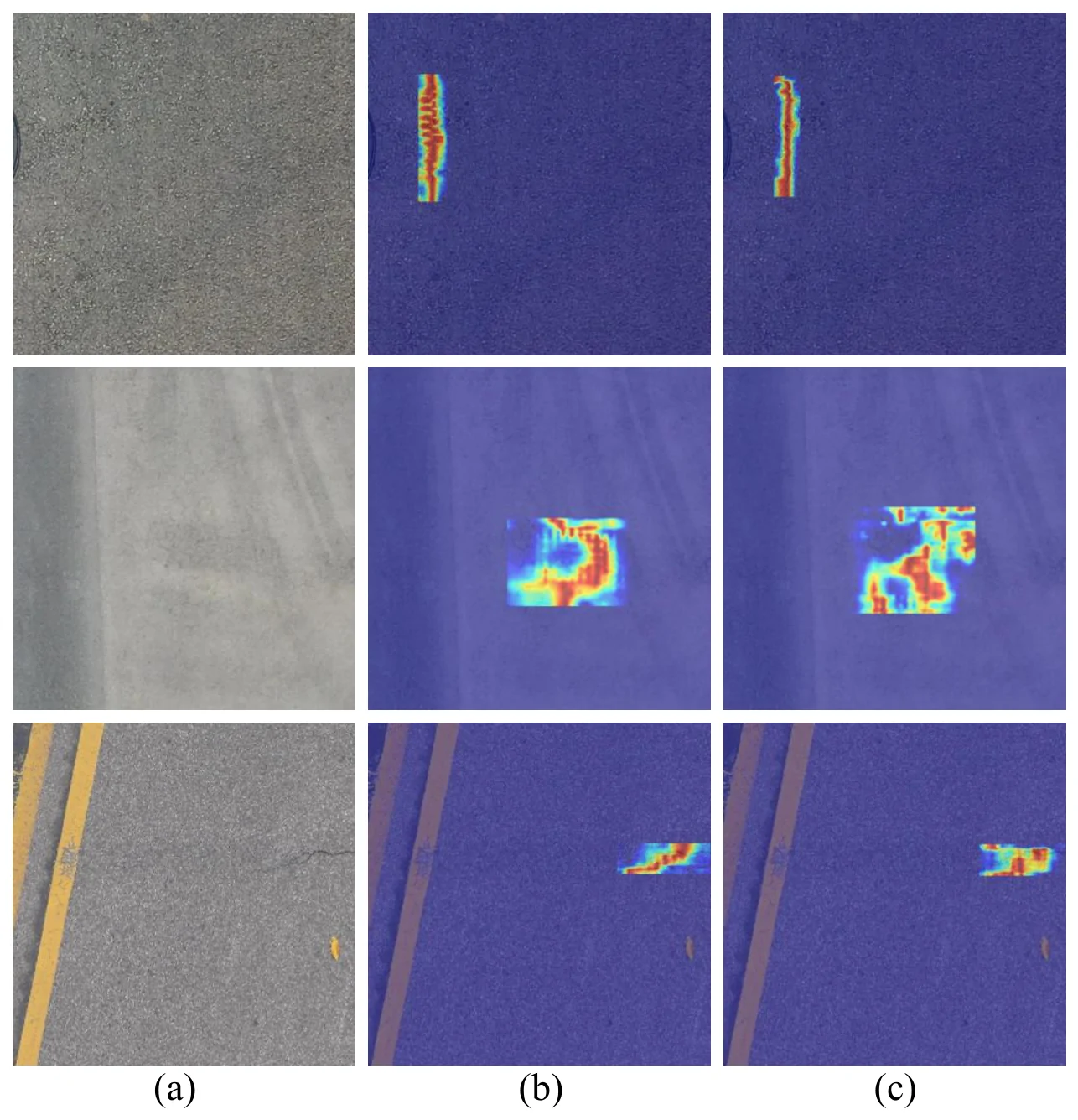
Qualitative assessment of detection performance: (**a**) unprocessed input images; (**b**) baseline YOLOv8n predictions; (**c**) results generated by the proposed BDC-YOLO.

In order to intuitively observe the perceptual differences and model fit on the visual level, the Grad-CAM technique is employed to compare the heat maps of the baseline model (YOLOv8n) and the proposed BDC-YOLO model, as illustrated in Figure 9. A heat map functions as a visual instrument, utilising continuous colour gradients to encode the distribution of attention and confidence levels within the detection network. The disparity between low and high feature activation values is spatially represented through cool and warm thermal tones, respectively. Specifically, a spatial gradient spanning from dark blue (indicating a low-attention background context) to bright red (designating high-confidence target regions) is applied to emphasise feature contrast.

As demonstrated in the comparative data, the baseline model frequently encounters difficulties in aligning with the authentic geometric morphology of road damages due to the constraints imposed by the rigid structure of standard square convolutions. For instance, in the third comparative pair, the baseline model’s activation area exhibits a broad and blocky distribution, resulting in the incorporation of a significant amount of irrelevant background pixels. In the second pair, which depicts complex alligator cracks, the baseline’s attention appears to be fragmented and blurry, leading to an inability to capture the fine-grained branch structures.

In stark contrast, the BDC-YOLO model evinces considerably elevated activation intensity and sharpness within the regions of interest (ROI). For linear damages (the first and third pairs), the high-saturation red areas of BDC-YOLO demonstrate continuous and exacting adherence to the irregular topological trajectories of the cracks, thus providing comprehensive evidence of the precision of the dynamic tubular receptive field provided by the DySnakeConv module. Concurrently, for mesh-like damages (the second pair), the enhanced model accurately captures the subtle crack networks while effectively preventing interference from background noise. These visual enhancements imply that BDC-YOLO possesses stronger feature extraction capabilities and increased anti-interference robustness, thus enabling it to more precisely understand diverse damage morphologies in complex pavement scenarios.

In conclusion, BDC-YOLO has exhibited favourable generalisation proficiency and localisation precision when confronted with a diverse array of road defects, ranging from substantial regional damage to minute linear cracks. The model has demonstrated its aptitude not only in robustly handling prevalent defects with discernible characteristics, but also in effectively addressing fine cracks with intricate morphologies. Consequently, it offers a dependable detection solution for pragmatic road inspection operations.

### 4.8. Qualitative Evaluation and Limitation Analysis

**Figure 10:**
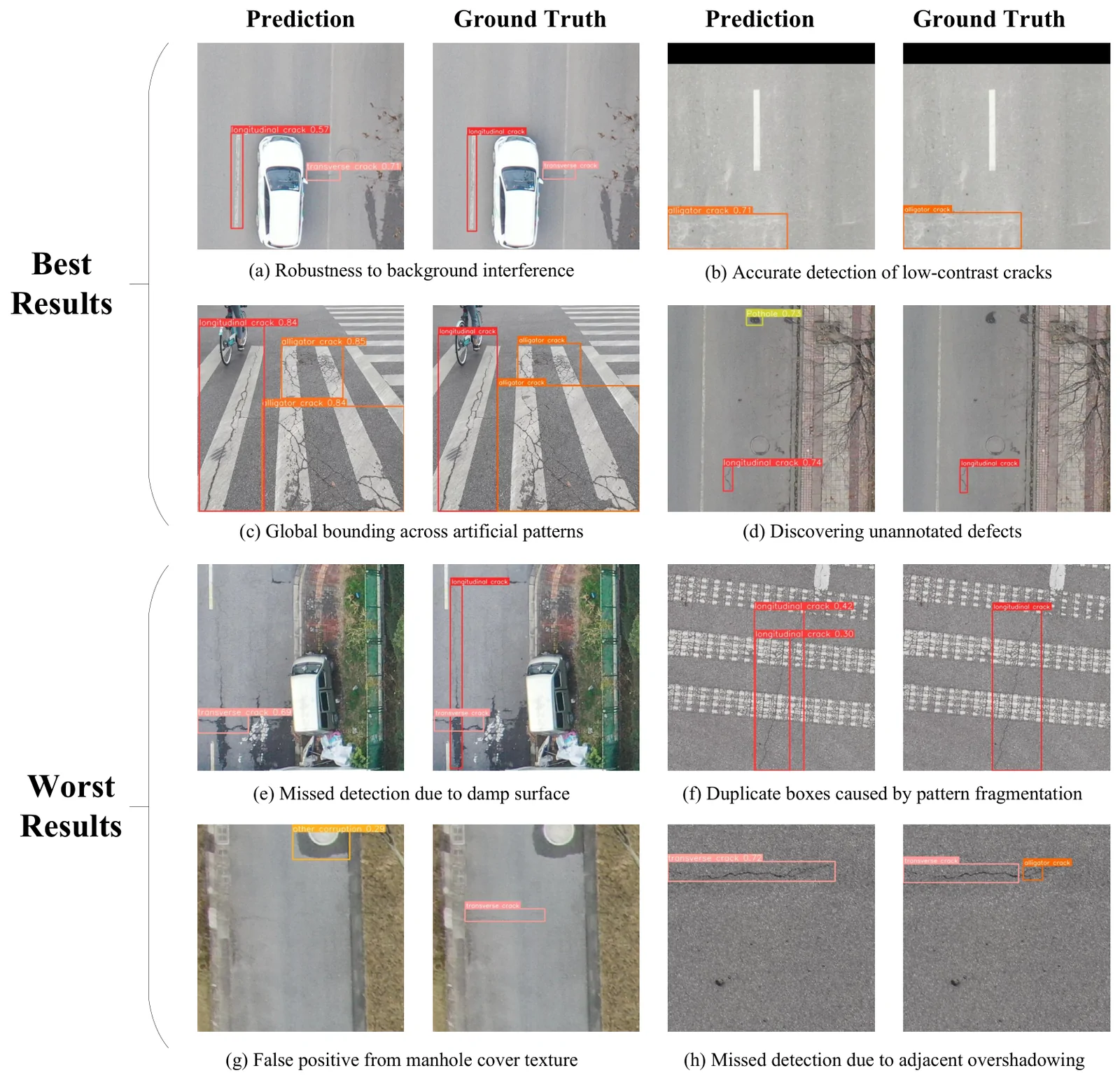
Qualitative evaluation of representative best (**a**–**d**) and worst (**e**–**h**) detection results.

To further evaluate the proposed BDC-YOLO model, a qualitative analysis was conducted using various real-world images. Figure 10 shows the selected visualizations. It includes successful detection results (Figure 10a–d) and typical failure cases (Figure 10e–h).

As shown in the top half of Figure 10, the model performs well in complex environments. First, it correctly separates targets from background noise, such as vehicle shadows. This reduces false positive predictions (Figure 10a). Second, the model successfully detects faint, low-contrast cracks that blend into the road surface (Figure 10b). Third, when facing artificial patterns like crosswalks (Figure 10c), the network connects broken crack segments effectively. It also ignores moving objects like bicycles. Finally, the model shows good robustness to labeling errors. It can find real road damages (e.g., potholes) that were missed by human annotators (Figure 10d).

However, the model still has some limitations under extreme conditions, as shown in the bottom half of Figure 10. First, severe water stains on the road greatly reduce image contrast. This occasionally causes the model to miss defects (Figure 10e). Second, thick white lines on crosswalks (Figure 10f) can visually break long cracks into pieces. This causes the model to generate duplicate bounding boxes for a single crack. Third, objects like manhole covers or road patches sometimes trigger false positives due to their confusing textures (Figure 10g). In these noisy areas, the model may also miss nearby faint cracks. Finally, when different types of defects are close together (Figure 10h), the strong features of a large defect can hide the weak features of a small one (such as a minor alligator crack). This also leads to missed detections.

Analyzing these failure cases provides an objective view of the model’s current boundaries. The results suggest that future work should focus on two aspects: improving feature extraction in low-contrast areas and better separating densely packed defects.

## 5. Evaluation of Generalization Capability

**Figure 11:**
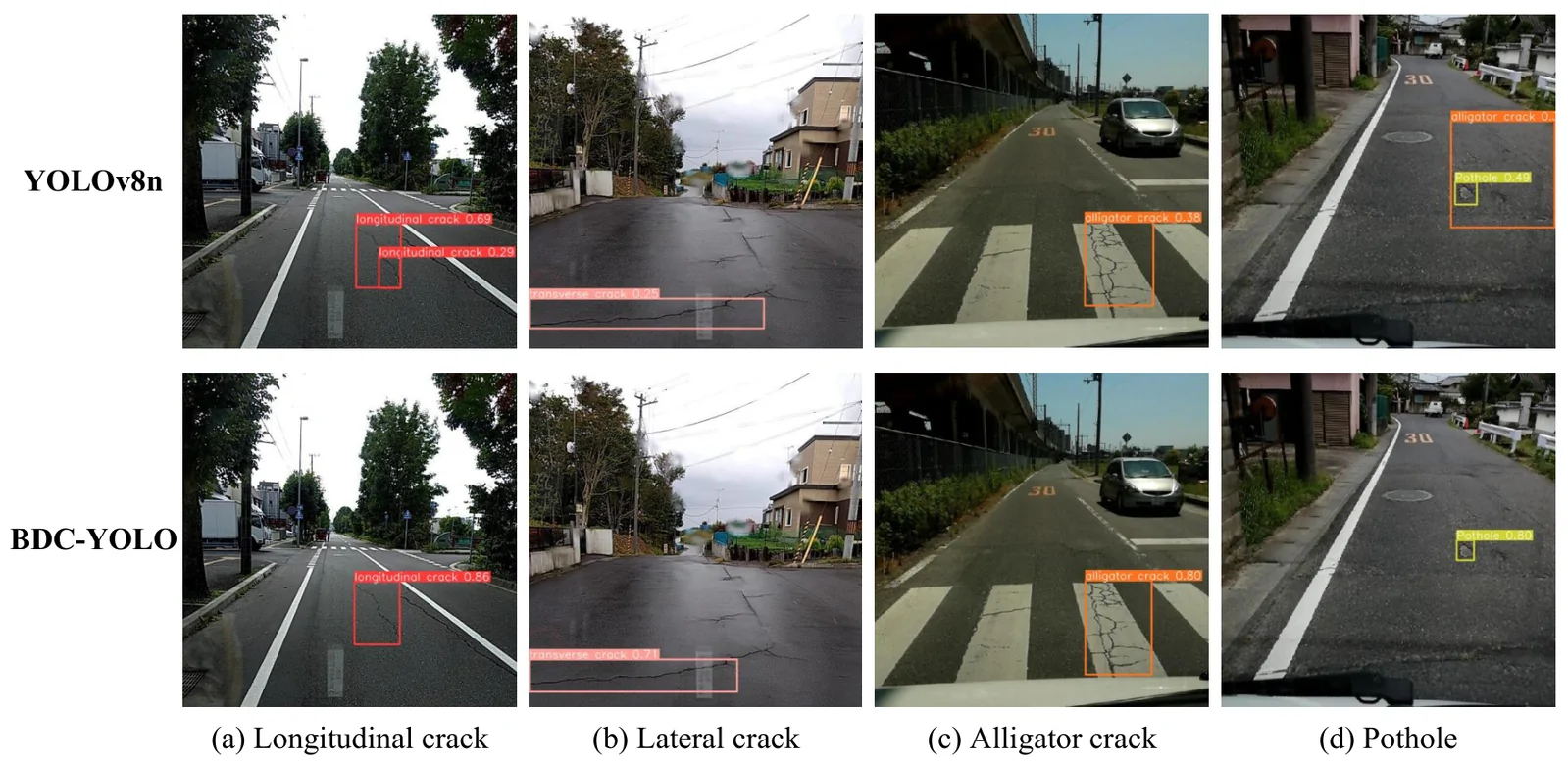
Qualitative comparison of pavement distress detection by YOLOv8 and BDC-YOLO using the Japanese subset of RDD2022.

Beyond the primary experiments on the RDDChina dataset, we further validated the cross-domain robustness of our model using an independent Japanese subset from the RDD2022 benchmark. Although sharing identical distress categories, this unseen dataset features distinct asphalt textures, lane markings, and illumination conditions. As demonstrated in Figure 11, the baseline YOLOv8 struggles to adapt to these unfamiliar road environments, frequently generating overlapping bounding boxes and exhibiting reduced confidence scores. In contrast, the proposed BDC-YOLO achieves stable and precise localization across various defect types, ranging from narrow linear cracks to irregular potholes and alligator cracking. By successfully suppressing regional background noise and accurately extracting target boundaries, the enhanced architecture maintains high detection accuracy on heterogeneous data. This cross-regional consistency demonstrates the algorithm’s applicability and reliability for deployment in diverse real-world engineering scenarios.

## 6. Conclusions

Constructed upon the baseline YOLOv8n architecture, the BDC-YOLO framework is formulated to resolve three fundamental bottlenecks in pavement damage detection: complex background interference, the irregular topological structures of cracks, and the loss of fine-grained features.

The Bi-level Routing Attention (BRA) is introduced as a semantic filter to establish a global receptive field, with the effect that high-frequency background noise such as road joints and tree shadows can be effectively suppressed. Subsequently, the DySnakeConv module is integrated to provide a dynamic tubular receptive field, which adaptively conforms to the morphological trajectories of irregular linear damages, thus avoiding the inclusion of irrelevant pixels caused by conventional square convolutions. Finally, the CARAFE module replaces the traditional nearest-neighbour upsampling to perform local content-aware feature reassembly, thereby thoroughly breaking the information transmission bottleneck within the Feature Pyramid Network (FPN) and ensuring the seamless alignment of multi-scale details.

A substantial body of experimental evidence has been collated which demonstrates the efficacy of BDC-YOLO in overcoming the mutual exclusivity of precision and recall. In comparison with the baseline model, a significant enhancement of 4.7 percentage points in the comprehensive metric, mAP_0.5_, was achieved, reaching 88.9%. The model displays remarkable robustness and precise localization capabilities, particularly in the identification of highly challenging targets such as alligator cracks and potholes, while maintaining an acceptable computational overhead.

Subsequent studies will concentrate on the implementation of the BDC-YOLO model on mobile edge computing devices, such as Unmanned Aerial Vehicles or vehicle-mounted cameras, for practical engineering applications. Further exploration will be undertaken into model lightweighting techniques, including knowledge distillation and parameter quantization. Furthermore, it is important to acknowledge the limitations of the current study regarding environmental generalization. The evaluation of the proposed BDC-YOLO model is primarily based on the RDDChina dataset, which predominantly consists of images captured during daytime under relatively favorable weather conditions. Consequently, the model’s robustness and detection accuracy under extreme weather (e.g., heavy rain, snow) or poor lighting conditions (e.g., nighttime, dense fog) have not been comprehensively verified. In our future work, we plan to construct a more diverse pavement dataset encompassing various weather and lighting scenarios. Additionally, we will explore integrating low-light image enhancement algorithms and multi-modal sensor fusion (such as combining RGB cameras with LiDAR or thermal imaging) to achieve robust, all-weather automated pavement inspection.

Moreover, while the current model focuses on intrinsic crack topologies to achieve generalization across standard asphalt and concrete pavements, verifying its cross-domain adaptability on fundamentally different road materials remains a critical next step. Therefore, our future research will concurrently explore domain adaptation techniques to extend the model’s robust inspection capabilities to diverse and non-standard road infrastructures, ultimately moving towards a truly universal pavement evaluation system.

## Figures and Tables

**Table 1:** Statistics of dataset.

Dataset	Image	Label	Resolution
ChinaDrone	2401	3068	512 × 512
ChinaMotorBike	1977	4650	512 × 512
RDDChina	3853	7718	512 × 512

**Table 2:** Experimental environment.

Category	Specification
Operating system	Ubuntu 20.04
Hardware environment	CPU: Intel CPU
	GPU: NVIDIA RTX 5070 Ti (16 GB VRAM)
	Memory: 32 GB
Software environment	Programming language: Python 3.10
	Deep learning framework: PyTorch 2.9
	CUDA version: CUDA 13

**Table 3:** Comparison results of different algorithms.

Model	P/%	R/%	mAP_0.5_/%	Params/M	GFLOPs	FPS
Faster R-CNN [31]	83.3	48.6	65.9	28.32	474.0	21.8
DETR-R50 [38]	48.7	66.2	79.8	41.50	57.11	92.4
YOLOv5n [39]	83.9	75.7	83.5	2.50	7.10	666.7
YOLOv7-tiny [40]	78.9	76.9	83.0	6.03	6.62	241.9
YOLOv8n [25]	84.5	75.6	84.2	3.01	8.10	526.3
YOLOv10n [41]	78.3	68.5	78.3	**2.27**	6.50	**1111.1**
YOLOv11 [42]	82.5	72.3	81.2	2.58	**6.30**	666.7
YOLOv26n [43]	80.0	71.7	79.9	2.47	6.70	909.1
OBC-YOLOv8 [19]	78.3	80.5	86.7	3.2	8.0	222
BDC-YOLO	**88.1**	**82.5**	**88.9**	4.09	10.60	175.4

Bold values indicate the optimal results among the comparison methods.

**Table 4:** Comparison of ablation experiments of the entire BDC-YOLO model.

BRA	DySnakeConv	CARAFE	P/%	R/%	mAP_0.5_/%	Params/M	GFLOPs	FPS
			84.5	75.6	84.2	**3.01**	**8.1**	**526.3**
✓			84.7	81.6	87.0	3.27	8.3	**526.3**
	✓		85.8	78.1	86.4	3.42	8.7	500.0
		✓	84.6	77.2	85.4	3.15	8.4	285.7
✓	✓		85.7	81.8	88.3	3.95	10.3	222.2
✓		✓	85.1	81.3	87.9	3.41	8.6	270.3
	✓	✓	84.9	81.1	87.2	3.82	10.3	212.8
✓	✓	✓	**88.1**	**82.5**	**88.9**	4.09	10.6	175.4

Bold values indicate the optimal results among the comparison methods.

**Table 5:** Ablation study of architectural configurations and design choices.

Method/Configuration	mAP_0.5_/%	P/%	R/%
*DySnakeConv Integration Strategy*			
Head only	86.6	84.4	81.5
**Backbone + Head (Ours)**	**88.3**	**85.7**	**81.8**
*BRA Parameter Setting (Region, Top-k)*			
[8, 7] (Default)	86.1	83.5	80.1
**[8, 4] (Ours)**	**87.0**	**84.7**	**81.6**

**Table 6:** Detection results of different road defects (YOLOv8n).

Class	mAP_0.5_/%	P/%	R/%
all	84.2	84.5	75.6
longitudinal crack	85.9	82.7	78.7
transverse crack	82.5	81.1	75.7
alligator crack	81.2	89.2	72.4
other corruption	86.2	82.3	77.5
pothole	85.3	87.0	73.6

**Table 7:** Detection results of different road defects (BDC-YOLO).

Class	mAP_0.5_/%	P/%	R/%
all	88.9	88.1	82.5
longitudinal crack	88.4	87.3	81.2
transverse crack	84.6	80.6	78.5
alligator crack	86.0	88.0	76.2
other corruption	89.9	88.6	86.0
pothole	95.9	96.2	90.4

**Table 8:** Comparison of the proposed mechanisms with representative alternative modules.

Replaced Module Stage	Alternative Mechanisms	P/%	R/%	mAP_0.5_/%
Attention	+CBAM	85.4	76.7	85.2
	+MPCA	82.3	77.3	85.2
	+CPCA	84.8	78.0	85.5
	**+BRA (Ours)**	**84.7**	**81.6**	**87.0**
Feature Extraction	+EIEM	82.2	76.3	83.3
	+Ghost	83.5	76.1	85.0
	**+DySnakeConv (Ours)**	**85.8**	**78.1**	**86.4**
Multi-scale Fusion	+AFPN	81.6	73.2	82.6
	+CAFM	84.6	74.9	84.4
	+BiFPN	83.2	75.4	85.1
	**+CARAFE (Ours)**	**84.6**	**77.2**	**85.4**

Bold values indicate the optimal results within each comparative group.

## Data Availability

Publicly available datasets were analyzed in this study. This data can be found here: https://doi.org/10.6084/m9.figshare.21431547.v1.
